# Fixation-related potentials during a virtual navigation task: The influence of image statistics on early cortical processing

**DOI:** 10.3758/s13414-024-03002-5

**Published:** 2025-01-23

**Authors:** Anna Madison, Chloe Callahan-Flintoft, Steven M. Thurman, Russell A. Cohen Hoffing, Jonathan Touryan, Anthony J. Ries

**Affiliations:** 1 U.S. DEVCOM Army Research Laboratory, Humans in Complex Systems, Aberdeen Proving Ground, MD USA; 2 Warfighter Effectiveness Research Center, Department of Behavioral Sciences & Leadership, 2354 Fairchild Drive, Suite 6, U.S. Air Force Academy, CO 80840 USA

**Keywords:** Fixation-related potentials, EEG, Eye tracking, Virtual environments, Unfold Toolbox, Image statistics

## Abstract

Historically, electrophysiological correlates of scene processing have been studied with experiments using static stimuli presented for discrete timescales where participants maintain a fixed eye position. Gaps remain in generalizing these findings to real-world conditions where eye movements are made to select new visual information and where the environment remains stable but changes with our position and orientation in space, driving dynamic visual stimulation. Co-recording of eye movements and electroencephalography (EEG) is an approach to leverage fixations as time-locking events in the EEG recording under free-viewing conditions to create fixation-related potentials (FRPs), providing a neural snapshot in which to study visual processing under naturalistic conditions. The current experiment aimed to explore the influence of low-level image statistics—specifically, luminance and a metric of spatial frequency (slope of the amplitude spectrum)—on the early visual components evoked from fixation onsets in a free-viewing visual search and navigation task using a virtual environment. This research combines FRPs with an optimized approach to remove ocular artifacts and deconvolution modeling to correct for overlapping neural activity inherent in any free-viewing paradigm. The results suggest that early visual components—namely, the lambda response and N1—of the FRPs are sensitive to luminance and spatial frequency around fixation, separate from modulation due to underlying differences in eye-movement characteristics. Together, our results demonstrate the utility of studying the influence of image statistics on FRPs using a deconvolution modeling approach to control for overlapping neural activity and oculomotor covariates.

**Supplementary Information:**

The online version contains supplementary material available at 10.3758/s13414-024-03002-5.

## Introduction

The human visual system evolved to be sensitive to statistical regularities within the external environment as an efficient way of processing information (Barlow, 1961). An important aspect of visual processing, called scene processing, underlies humans’ ability to perceive the external environment and form an internal representation that provides context to visual information as it propagates through the visual pathway (Oliva & Torralba, 2001). Our ability to form scene representations results from elementary visual features from edges and surfaces in the environment, such as contrast, spatial frequency, color, and orientation (Groen et al., 2017).

Using electroencephalography (EEG) and event-related potentials (ERPs), early visual processing in the magnocellular layers, sensitive to low spatial frequency stimuli, and parvocellular layers of the lateral geniculate nucleus, sensitive to high spatial frequency stimuli, occurs at slightly different time courses (Ellemberg et al., 2001; Hansen et al., 2011, 2012; Ries & Hopfinger, 2011). Consistent with the organization of early visual pathways, theoretical frameworks posit that scene processing occurs in a coarse-to-fine manner, where following the visual hierarchy, lower spatial frequency information first influences scene representations followed by higher spatial frequency information (Groen et al., 2013; Hegde, 2008; Kauffmann et al., 2014, 2015; Petras et al., 2019; Schyns & Oliva, 1994). It has been shown that during viewing of complex real-world scene images, neural representations fluctuate prioritizing different spatial frequencies along the visual hierarchy in time (Hansen et al., 2021).

Most experimental evidence on scene perception comes from paradigms using images of complex scenes from the real-world which allows researchers to compute image statistics or properties (Groen et al., 2013, 2017; Hansen et al., 2003, 2021; Hansen & Hess, 2006, 2007; Henderson et al., 2020; Henderson & Hayes, 2017; Oliva & Torralba, 2001; Seijdel et al., 2020). Natural scenes are broadband, meaning they contain a spectrum of spatial frequencies that constitute the different textures and objects present in a given image. Physical regularities within natural scenes or images follow an established pattern where contrast energy at different spatial frequencies (*f*) falls with increasing spatial frequency following a 1/*f*^α^ relationship (Billock, 2000; Field, 1987; Hansen et al., 2003, 2021; Hansen & Essock, 2005; Hansen & Hess, 2006; Tolhurst et al., 1992; Van der Schaaf & Van Hateren, 1996). Given an image, α can be measured as the slope of the orientation averaged amplitude spectrum obtained from the Fourier transformation of that image, and when plotted on a logarithmic scale α approaches 1.0 (Tadmor & Tolhurst, 2000; Tolhurst et al., 1992).

Leveraging this characteristic of natural scenes or images, studies using EEG have either measured image statistics or artificially manipulated them to measure changes in visually evoked potentials and ERPs (De Cesarei et al., 2013; Groen et al., 2017, 2018; B. C. Hansen et al., 2011, 2012; B. C. Hansen & Hess, 2007; Rokszin et al., 2016). This work has led to the understanding that the P1 and N1 components of the visually evoked potential code for different aspects of spatial frequency, luminance, and contrast (B. C. Hansen et al., 2011, 2012). Extending this work, the P1, N1, and P2 components of the visual ERP can distinguish between scene texture (Balas & Conlin, 2015) and global scene properties, such as openness and spatial expanse (N. E. Hansen et al., 2018; Harel et al., 2020).

By and large, this body of work has taken calculated departures from naturalistic visual conditions to isolate and understand visual processing under a controlled context by limiting eye movements. However, while restricting eye movements in these studies limits confounds from underlying differences in eye movements (Nikolaev et al., 2016) and prevents ocular artifacts in the EEG recording, it does so at the cost of minimizing how visual information is typically selected—through directed eye movements. Since most of what we know about early visual evoked potentials, such as the P1 and N1, have followed the traditional EEG approach, little is known about how these response characteristics might translate to free-viewing experiments.

### The fixation-related potential approach

Co-recording of eye-tracking and EEG data provides a means to use naturally occurring eye events, such as fixation or saccade onsets, as time-locking events in the EEG recording. Importantly, this approach overcomes limitations of traditional EEG experimental paradigms by allowing investigation of visual processing during natural eye behaviors. Time-locking the EEG data to fixation onsets results in fixation-related potentials, or FRPs, and provides a neural snapshot to study visual processing. There is a growing body of FRP experiments leveraging this co-recording approach to study aspects of visual processing, such as the parafoveal preview in reading (Antúnez et al., 2022; Mirault et al., 2020), object and scene processing (Coco et al., 2020), face processing (Buonocore et al., 2020; Huber-Huber et al., 2019), visual search (Brouwer et al., 2013, 2017; Kaunitz et al., 2014; Nikolaev et al., 2013; Nuthmann & Canas-Bajo, 2022), change detection (Nikolaev et al., 2011), and memory (Nikolaev et al., 2023a). This is largely due in part to advances in data synchronization and signal processing methods, such as independent component analysis (ICA) to minimize ocular artifacts (Dimigen, 2020; Plöchl et al., 2012).

But due to the rapid nature of vision, free-viewing paradigms lead to difficulty in parsing neural responses elicited by any one fixation because temporally adjacent fixation events can cause overlap of the two FRPs (Dimigen et al., 2011; Nikolaev et al., 2016). Indeed, recent evidence suggests multiple visual representations can co-exist simultaneously within the visual hierarchy, where neural activity from a given stimulus can persist for longer than stimulus presentations lasting longer than 1 s (Grootswagers et al., 2019; King & Wyart, 2021; Robinson et al., 2019). An approach to overcome this overlap issue is to use deconvolution modeling to correct for ongoing activity that might overlap with each other, such as saccade and fixation-related activity (Dimigen & Ehinger, 2021; Ehinger & Dimigen, 2019; Smith & Kutas, 2015a). Together, signal and data processing advances can directly address data analytic challenges that arise from co-recording of eye tracking and EEG data.

### The lambda response

A well-established FRP component called the lambda response reflects afferent visual input to visual cortex and is characterized by a positive deflection peaking around 80 ms after fixation onset over occipital electrodes (Oz, O1, and O2) when referenced to the average mastoids (Billings, 1989; Thickbroom et al., 1991; Yagi, 1979). A prominent finding in the FRP literature is the magnitude of the preceding saccade impacts the amplitude of the lambda response (Dandekar et al., 2012; Ries et al., 2018; Thickbroom et al., 1991). Evidence from several studies suggests the relationship between saccade magnitude and the lambda response amplitude is nonlinear. Some studies have found smaller saccades lead to linear increases in the lambda response amplitude (Dimigen et al., 2011), whereas others have found the increase in saccade size saturates around 11°, resulting in a decrease in amplitude with saccades larger than 12° (Nikolaev et al., 2016; Ries et al., 2018). Evaluating the lambda response under dark lighting conditions suggests that this influence of saccade size on the FRP amplitude may not be completely due to visual stimulation (Dimigen, 2021). Since the FRP can be influenced by eye-movement characteristics (Yagi, 1979), previous studies have circumvented this issue by artificially controlling for eye-movement characteristics, such as fixation durations (Brouwer et al., 2013; Kaunitz et al., 2014) or saccade magnitudes (Kazai & Yagi, 2005; Ries et al., 2016; Touryan et al., 2017) to control for low-level influences that may differ between experimental conditions.

In addition to saccade magnitude, the lambda response has been shown to be influenced by both bottom-up visual features, such as contrast (Kazai & Yagi, 1999, 2005), luminance (Ossandón et al., 2010), and spatial frequency (Ries et al., 2018; Yagi et al., 1993), as well as top-down goals (Ries et al., 2016). This follows, since the lambda response is believed to be analogous to the P1 from traditional ERP studies due to evidence suggesting both components have a common neural generator in striate or extrastriate cortex (Kazai & Yagi, 2003). Varying the contrast and spatial frequency of black-and-white sinusoidal gratings, Kazai and Yagi (2005) found the contrast dependence of the lambda response depended on the spatial frequency of the stimulus. They found that the lambda response amplitude did not change with varying contrast below 1 cycle(s) per degree (cpd; i.e., in the 0.5 cpd and 1 cpd conditions), but above 1 cpd (i.e., in the 3 cpd condition) had a larger amplitude to medium contrasts. Using Gabor patches that varied in spatial frequency, Ries and colleagues (2018) found that the lambda response amplitude was larger for lower spatial frequencies (e.g., 1.5 cpd and 3 cpd) compared with higher spatial frequencies (e.g., 12 cpd). 

Kazai and Yagi (1999) posited that FRP activity is influenced by activity across fixations. To explore this idea, they looked at changes in the lambda response and FRP N1 using checkboard stimuli across fixations, where the checkerboard pattern could be different sizes or could change from one fixation to the next in terms of pattern size or phase. They found that two fixations, where the checkboard pattern reversed from a small to a large pattern size, resulted in a larger lambda response amplitude compared with when the checkerboard pattern stayed the same or went from a large to a small pattern size. Additionally, Kazai and Yagi (1999) looked at the influence of the N105, or the N1, and that going from a large to smaller checkerboard pattern resulted in a larger N1 amplitude. Although this work did not directly measure spatial frequency, it suggests that changes in stimuli spatial frequency across fixations might influence early components of the FRP. Following the evaluation of consecutive fixations, Ossandón and colleagues (2010) used grayscale natural scene images to investigate the influence of luminance on the FRP. Comparing the activity between consecutive fixations, they found that changes in absolute luminance between fixations led to larger lambda responses.

Similar to traditional EEG experiments, many of the published studies on FRPs present overly simplified and/or static stimuli. So, while the influence of saccade size, luminance, and spatial frequency are known to affect lambda response amplitudes, it is unknown if these same findings hold in in the presence of complex, free-viewing paradigms presented in color. The divergence between fixed-gaze setups to maintain experimenter control and natural eye behavior begs the question of whether prior findings from highly controlled experimental setups from both traditional ERP and FRP approaches translate into more naturalistic contexts. More specifically, are the lambda response and N1 FRPs independently modulated by luminance and spatial frequency when controlling for saccade magnitude? If so, are these FRP components affected by changes in image statistics from fixation to fixation?

### The current study

In the present study, we co-recorded eye tracking and EEG while participants completed a visual search and navigation task in a complex virtual environment presented on a computer monitor. Here, we focus on how low-level image statistics influence early visually evoked potentials, both the lambda response and N1, after fixation onsets. Our approach leverages the use of virtual environments to simulate a real-world outdoor environment that is continuous, complex, and dynamically changes with participants’ position in space. A main benefit to this approach is that it allows us to compute post hoc image statistics related to each participant’s fixation locations and to co-register fixations to objects in the scene. Importantly, we use advances in recent data processing techniques to complement the co-registration of EEG and eye movement data by optimizing the removal of ocular artifacts through an independent component-based approach (Dimigen, 2020) and deconvolution modeling to control for overlapping neural activity and low-level signal variability (e.g., saccade size and fixation duration; Ehinger & Dimigen, 2021). We show that the FRP is influenced by low-level image statistics, such as luminance and a relative metric of spatial frequency, when controlling for the influence of saccade magnitude.

## Methods

### Participants

Thirty-eight participants were recruited through an online questionnaire (used as a prescreen) and invited to take part in a study at the U.S. Army Research Lab (ARL) West in Playa Vista, CA. Participants gave informed consent prior to starting the experiment via a written form approved by the ARL’s Institutional Review Board (ARL 19–122) and in compliance with the Declaration of Helsinki. Participants were at least 18 years or older and were an average age of 39.36 years (*SD* = 14.78, range: 19–64), with an average education of 15.94 years (*SD* = 2.33). All participants reported normal hearing and vision, were not susceptible to motion sickness, and had no prior brain injury. Overall, 27 participants were right-handed and reported using video games on average 1.97 h (*SD* = 1.26) a week. The experimental session took about 2.5 h, and participants were remunerated for their time. Participants had at least 20/40 vision (or better) and normal color vision, which was verified in the lab by using a Snellen Chart (visual acuity) and the Ishihara Test 14-plate version (color vision deficiency) prior to starting the experiment. Data from 33 participants (11 women) were used for the reported analysis. Data from five participants were excluded for the following: poor eye-tracking data quality (*n* = 2) or if the peak of the first positive deflection in the subject’s grand FRP at electrode Oz, the known maximum of the lambda response (Billings, 1989; Yagi, 1979, 1981), did not occur between 50 and 150 ms after fixation onset (*n* = 3).

### Apparatus

Participants completed all experimental tasks on a Corsair PC running Windows 10 with a custom Unity 3D project (2018.3.13f1, Unity Technologies) using a wired mouse and keyboard. All visual stimuli were presented full-screen on a Tobii Pro Spectrum Monitor (EIZO FlexScan EV2451) with a refresh rate of 120 Hz and resolution of 1,960 × 1,080 pixels. Participants were asked to maintain an upright, comfortable position seated approximately 70 cm from the display with no chin rest to restrict head position. On average, participants eye position was 61.94 cm (*SD* = 4.40 cm) from the eye tracker, a metric provided in the eye-tracker output resulting in a display visual angle of approximately 45° horizontally and 25° vertically.

### Study design and procedure

The data reported in this paper are a subset of a larger study aimed to investigate aspects of visual search and memory processes utilizing concurrent recording of physiological measures: eye movements, pupillometry, EEG, and electrocardiography (EKG). To this aim, participants complete three experimental tasks: (1) go/no-go rapid serial visual presentation task (memory task study phase), (2) visual search and navigation task, and (3) old/new recall task (memory task test phase). Here, we present an analysis focused on combined eye tracking and EEG data collected during the visual search and navigation task to evaluate FRPs related to scene processing. Other published papers focus on describing aspects of the eye movements and navigation task behavior (Enders et al., 2021) and pupillometry data (Thurman et al., 2021).

### Navigation and visual search task

Participants learned the virtual navigation task through a short tutorial. Specifically, participants practiced navigating using the keyboard (W, A, S, D keys) and how to change the current view of the virtual environment by moving the mouse. Next, participants began the search task by looking for and counting target objects (15 total) dispersed randomly throughout the virtual environment. The search task utilized a between-subjects design where participants were put into one of four target conditions: motorcycle (*n* = 13), Humvee (*n* = 9), furniture (*n* = 6), and aircraft (*n* = 5). Data collection was disrupted due to COVID, and as a result, there were fewer participants in the aircraft and furniture target conditions.

**Fig. 1:**
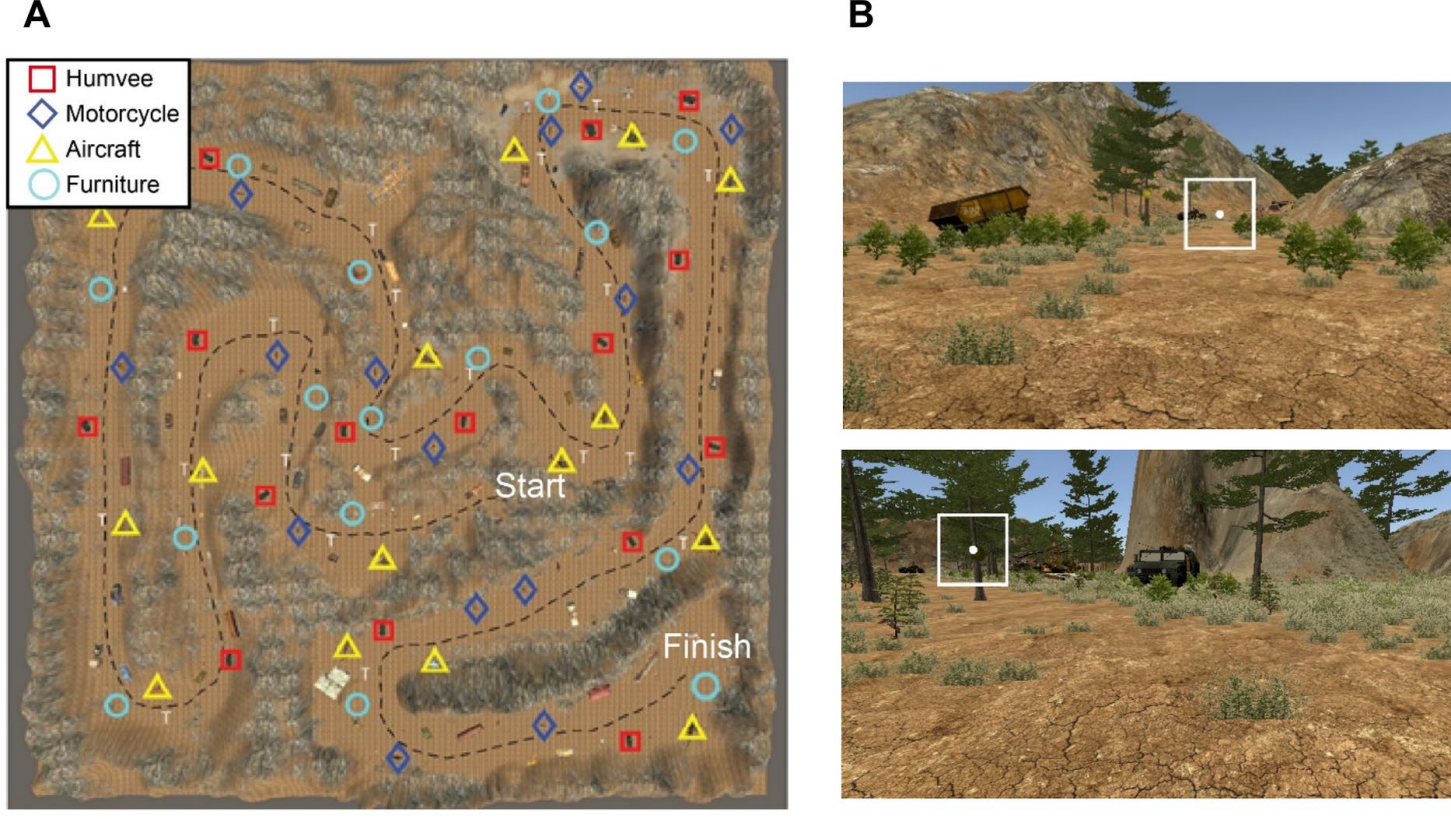
Overview of virutal environment. **A** Overview of navigation task enviornment with the target objects locations shown for each of the four target categories: motorcycle, Humvee, furniture, and aircraft. **B** An example of what participants saw in the virtual environment with the 5° patch shown centered on the fixation location used to calculate image statistics. (Color figure online)

Participants had agency to navigate freely through the virtual open world environment but followed trail markers, akin to signage found on a hiking trail, which provided direction through the virtual environment. Additionally, environmental features, such as trees and valleys, indicated path direction. Figure 1 shows a top-down and first-person view of the virtual environment. On average, it took participants 14.82 min (*SD* = 2.95) to complete the navigation task. While traversing the virtual environment and searching for target objects, an auditory math task occurred three times between 5 and 10 min into the task (~ 8 min on average) to increase cognitive load. The math task consisted of the aural presentation of three to four digits between the numbers of 0 to 9. Participants were required to sum up the three to four digits. After a tone occurred, participants had to report the sum of the series of numbers. The data analysis presented below *excludes* fixations occurring during the math task. For more information on the navigation and visual search task and how eye movements were influenced by target objects, see Enders et al. (2021).

### EEG and eye-tracking data recording

The EEG data were sampled at 2048 Hz from 64 active scalp electrodes using the Biosemi Active Two system (Amsterdam, Netherlands). Additional electrodes were placed on the left and right mastoids, at the outer canthus of each eye, and above and below orbital fossa of the left eye. All electrode impedances were below 30 kΩ and referenced to the Common Mode Sense (CMS) active electrode during the recording. A Tobii Pro Spectrum eye tracker was used to record participants’ eye movements and sampled at 300 Hz. The Tobii Pro Spectrum’s has an average binocular accuracy of 0.3° with spatial precision (root mean square) of 0.07° (Tobii Pro, 2018). Prior to the navigation task, a 5-point eye calibration was completed.

### Data processing

**Fig. 2:**
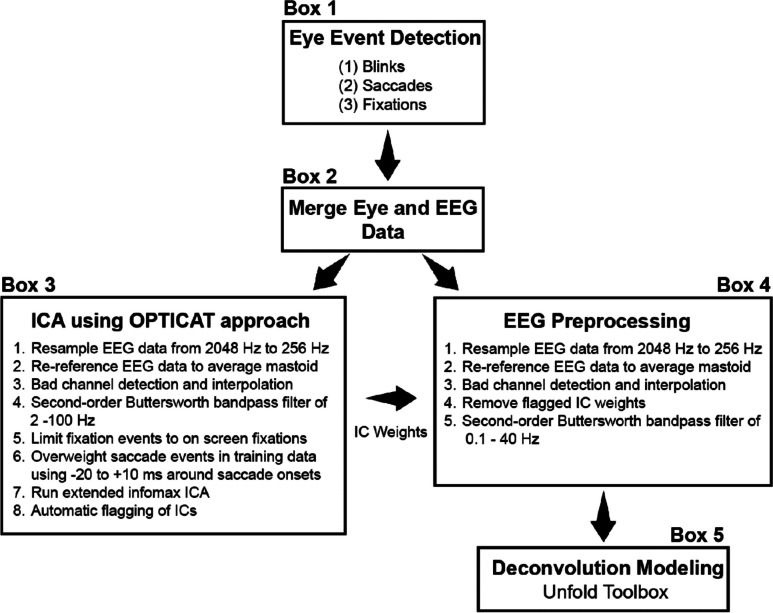
Overview of eye and EEG data processing pipeline. *Note.* Eye events were first detected (Box 1) and then merged with the raw EEG data (Box 2). The EEG processing demonstrates the parallel processing of the raw EEG data for ICA computation using the OPTICAT approach (Box 3) and for the deconvolution modeling using the Unfold Toolbox (Box 4 and 5)

Data from Unity, Tobii, and BioSemi system were synchronized through Lab Streaming Layer (LSL). LSL is an open-source research tool for unifying time-series data from different sensor and computer streams, which allows for the parallel recording of data streams from multiple different sources (Kothe et al., 2014). Data synchronization using LSL occurs through a process that relies on a clock offset measurement and a timestamp from each sample in every data stream recorded with the actual stream data. Subsequent data synchronization of the EEG, eye tracking, and Unity data was done offline. Figure 2 shows an overview of the steps in the data processing pipeline for the eye tracking and EEG data. The eye events were first detected, (Box 1) followed by the synchronization of eye and EEG data. Eye events were then added (i.e., blinks, saccades, and fixations) to the EEG data structure as unique events. The eye and EEG data were processed first at the subject-level using custom scripts in MATLAB (The MathWorks), and then aggregated for group-level statistical analysis using R (R Core Team, 2021) and RStudio (RStudio Team, 2021).

#### Eye-movement data

Eye events were detected offline from the Tobii eye-tracking data. First, blinks were detected by identifying stereotypical gaps of 50 to 500 ms in the gaze behavior (Holmqvist et al., 2011); gaps in data outside this duration were considered a dropout in the data. We then detected saccades and fixations events using a velocity-based algorithm (Engbert & Kliegl, 2003; Engbert & Mergenthaler, 2006) adapted from the EYE-EEG plugin (Dimigen et al., 2011). Saccade events were detected by calculating individual subject velocity thresholds for horizontal and vertical components separately using the median of the velocity time series smoothed over a five-sample window. Specifically, we used a velocity threshold of six (or six times the median velocity for each subject) and a minimum saccade duration of 12 ms to detect saccades. Fixations were defined as eye data that fell below this velocity threshold and were at least 50 ms in duration, and saccade events were not combined when fixation durations fell below this threshold. In effort of minimizing the amount of overlap between eye events, we only considered fixations between 100 and 2,000 ms in duration that were preceded by a saccade with (1) a duration between 12 and 100 ms, (2) an amplitude of 1° to 30°, and (3) a velocity between 30° and 1,500°/s for further analysis.

### Image statistics: Scene luminance and amplitude spectrum slope

**Fig. 3:**
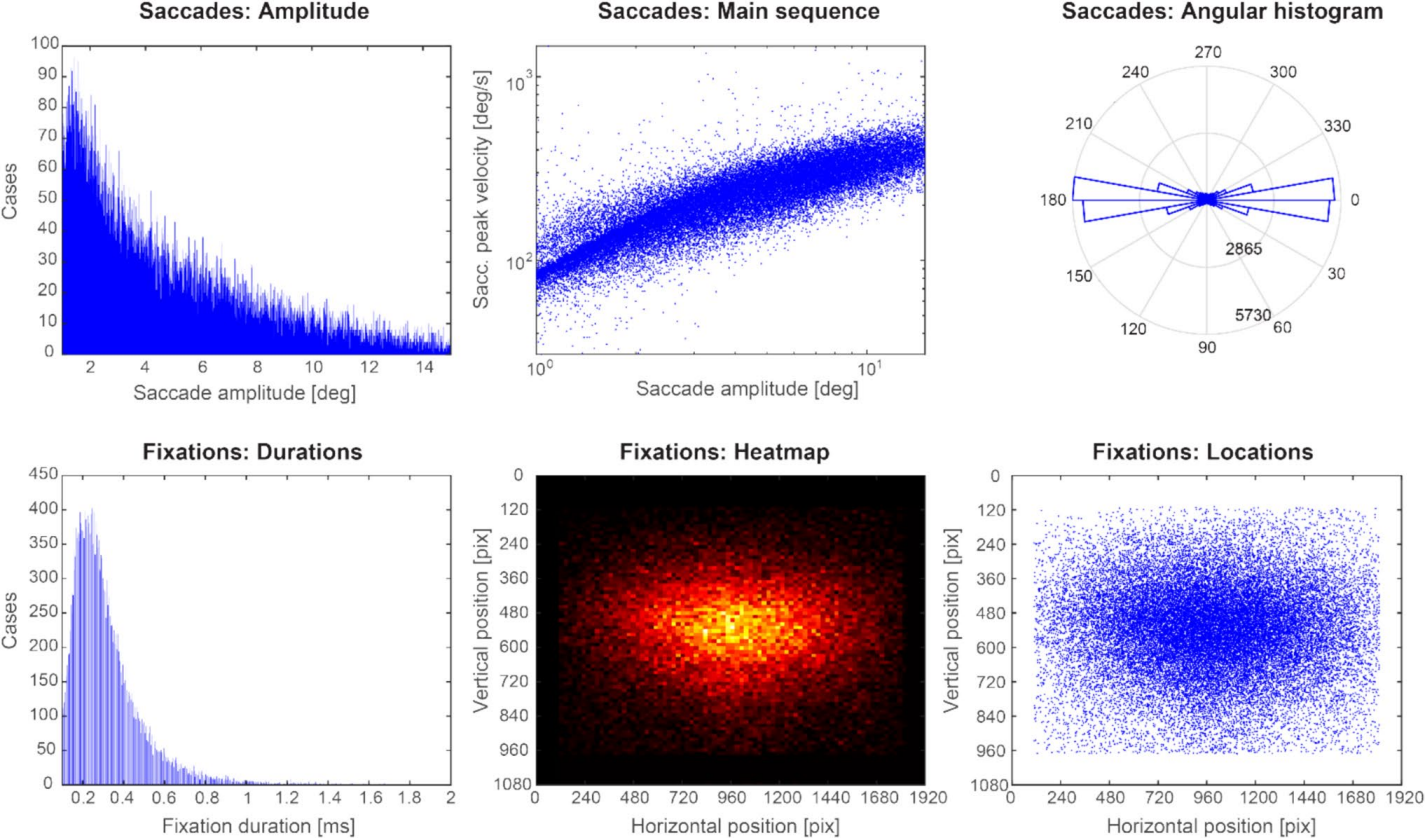
Descriptive plots of eye events used in the deconvolution modeling analysis. *Note.* Top row: The overall distribution of saccade amplitudes, main sequence showing the expected relationship between peak saccade velocity and saccade magnitude (Bahill et al., 1975), and histogram of saccade angles showing a larger bias for horizontal eye movements aggregated across all participants. Bottom row: The distribution of fixation durations and overall location of fixations during the navigation task showing a bias for the center of the computer monitor. (Color figure online)

Using a virtual environment affords the ability to recreate each participant’s unique experience during the navigation task by recording the virtual camera position during the navigation task. This allowed us to generate images (or snapshots) from the virtual environment that corresponded to each frame of the task and calculate post hoc image properties (screen refresh rate average = 120.78 Hz and *SD* = 0.064 Hz). For each participant, we first found the images presented on-screen that corresponded closest to fixation onset times. Next, we used the participant’s fixation location on the image (*x*, *y* pixel) from the left eye to create a 5° square patch centered on fixation. A 5° patch was chosen to be analogous to the size of the fovea and parafoveal region (Strasburger et al., 2011). This 5° patch was used to subsequently calculate image statistics for both luminance and scene spatial frequency. Figure 1B shows examples of snapshots with a 5° patch centered on fixation location used in the final FRP analysis. If a fixation location was too close to the edge of the computer monitor such that the 5° patch would fall “off the screen,” it was not used for calculating image statistics or used in the final analysis. This resulted in a data loss of 2.9% of the eye-movement data. Across the 33 participants, 41,370 fixations were used for the analysis with on average 1,011.21 fixations (*SD* = 310.73, range: 373–1,828) per subject. Depicted in Fig. 3 are descriptive plots of the eye-movement data used in the deconvolution modeling across all participants.

#### Luminance

Luminance for each color channel was empirically measured from the computer monitor using a spectroradiometer and gamma corrected following the procedure outlined by Thurman et al. (2021). This was accomplished using custom MATLAB code that systematically presented each RGB color channel from 0 to 255 in increments of three on the full screen, while the spectroradiometer measured luminance (cd/m^2^) at a distance equal to the participant’s distance from the screen. The screen luminance data were fit with an exponential function to estimate the best gamma parameter to create a color look-up table for transforming a given color channel pixel value into a luminance value (red gamma = 2.24, green gamma = 2.23, and blue gamma = 2.22). Average luminance was then calculated for 5° patches centered on fixation at the location of the fixation onset. Specifically, the average luminance was calculated by first computing the sum across the RGB color channels for each pixel to compute pixel-wise overall luminance, and then taking the average across all the pixels within the 5° patch around fixation. This resulted in a metric we refer to as the average luminance in our analysis.

#### Amplitude spectrum slope

Spatial frequency (*f*) from a natural image can be approximated and transformed into an amplitude spectrum that typically follows 1/*f*^α^ (Field, 1987; B. C. Hansen et al., 2003; B. C. Hansen & Hess, 2006; Tolhurst et al., 1992; Van der Schaaf & Van Hateren, 1996). The slope of the amplitude spectrum, or α, plotted on a log–log scale provides a relative metric of scene spatial frequency that can be compared across different frames of the virtual environment participants saw during the navigation task. We calculated α using MATLAB and the Image Processing (Version 10.4) and Signal Processing Toolboxes (Version 8.2). First, we converted each image used to calculate average luminance to grayscale. Next, we extracted the pixels corresponding to the 5° patch centered on the fixation location of the left eye, and added a Gaussian window with a standard deviation of π/2 centered on the fixation location. A two-dimensional Fourier transform was done on the 5° patch, and the zero-frequency component was shifted to the center. The amplitude spectrum was extracted using the square root of the power spectrum. Next, we took the average across all orientations, plotted this on a log–log scale, and fit a linear function to calculate the slope value representing α. A smaller slope of the amplitude spectrum (e.g., − 0.9) is associated with greater contrast energy at high spatial frequencies, and vice versa, a larger slope of the amplitude spectrum value (e.g., − 1.4) is associated with greater contrast energy at lower scene spatial frequencies.

#### Electroencephalographic (EEG) data

The EEG and subsequent FRP data were processed using EEGLAB (Delorme & Makeig, 2004), ERPLAB (Lopez-Calderon & Luck, 2014), EYE-EEG (Dimigen et al., 2011), and Unfold Toolboxes (Dimigen & Ehinger, 2021; Ehinger & Dimigen, 2019). Boxes 3 and 4 in Fig. 2 shows how we processed the EEG data separately for performing ICA to identify independent components (ICs) associated with ocular artifacts following the OPTICAT approach (Dimigen, 2020) and for preprocessing for the deconvolution modeling.

#### Correction for ocular artifacts

During free-viewing tasks, sources of ocular artifacts can arise from movement of the eyeballs, eyelids, and extraocular muscles that contaminate the neural activity (Plöchl et al., 2012). To identify activity in the EEG recording related to ocular artifacts, we performed an ICA on the EEG and electrooculogram (EOG) data using the extended Infomax algorithm (Lee et al., 1999) following the OPTICAT approach (Dimigen, 2020). The OPTICAT procedure optimizes the removal of eye muscle-generated saccadic spike potentials (Boylan & Doig, 1989; Keren et al., 2010; Plöchl et al., 2012) through more liberal filter settings to allow higher frequency activity to be retained in the training data, overtraining on saccade onset events, and using an automated process for flagging and rejecting ICs.

**Fig. 4:**
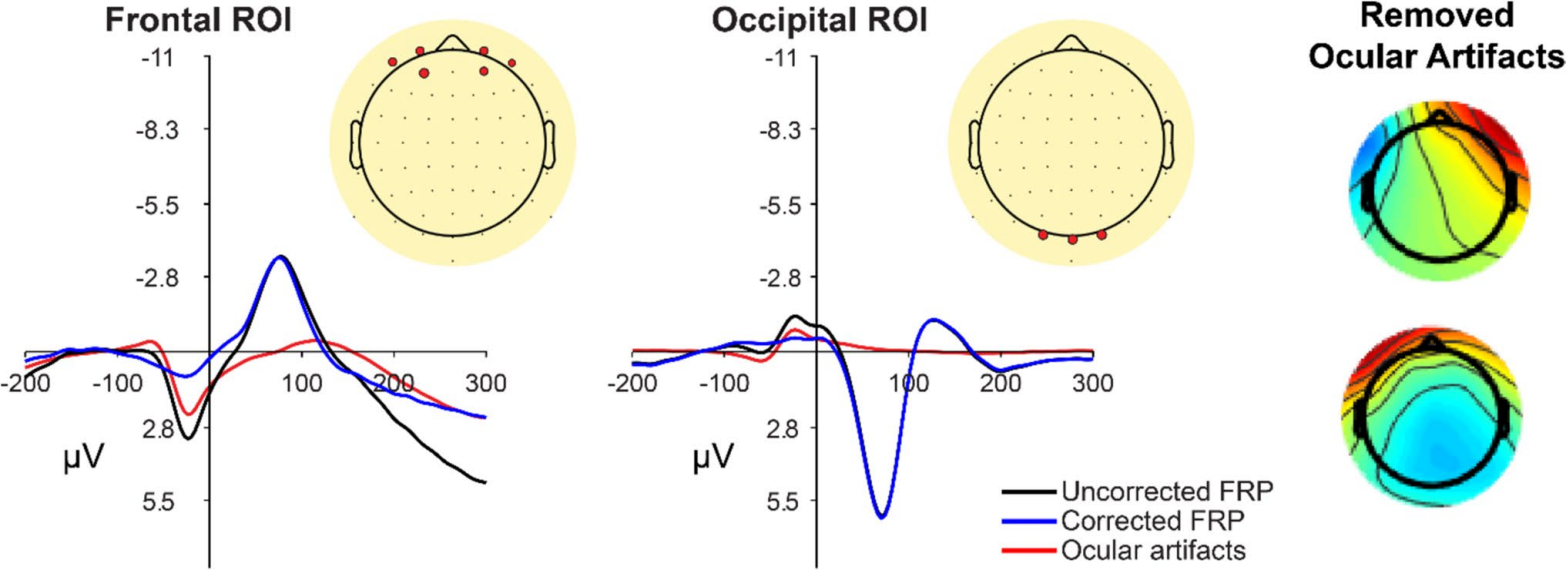
Impact of ocular artifact correction on the grand average FRP. *Note.* The group averaged FRP is plotted with positive down at a frontal ROI (Fp1, AF3, AF7, Fp2, AF4, AF9) and occipital ROI (O1, Oz, O2) electrodes with uncorrected EEG (black), isolated flagged ICs (red), and the resulting FRP once the ICs are removed (blue). Next to the FRPs are the corresponding topographic distribution of the typical flagged IC activity removed from one participant’s data. (Color figure online)

For each participant, the 64 channel EEG data were first resampled from 2048 to 256 Hz and re-referenced to the average of the left and right mastoids (Devillez et al., 2015; Fischera et al., 2013; Kaunitz et al., 2014; Nikolaev et al., 2013; Ossandón et al., 2010). We detected bad channels using a voltage threshold of four standard deviations (function: *pop_rejchan*) and then spherically interpolated them (function: *pop_interp*). Next, we filtered the EEG data using a second-order Butterworth filter with a bandpass filter from 2 to 100 Hz, based on previous work showing these settings are optimal for free-viewing scene perception paradigms (Dimigen, 2020). Prior to submitting the EEG data to ICA, we overweighted saccade events using EEG data occurring − 20 and + 10 ms from saccade onsets, resulting in twice the original event data. After running ICA, we used an automatic IC flagging/rejection process to classify activity related to ocular artifacts based on a procedure from Plöchl et al. (2012) implemented in the EYE-EEG toolbox (function: *pop_eyetrackerica*). We computed variance ratios between saccade epochs and fixation epochs (i.e., variance_saccade_/variance_fixation_) using a saccade window starting 11.7 ms (or 3-samples) before saccade onset until the saccade offset and fixation windows defined as from fixation onset to 11.7 ms before a saccade. If the mean variance of saccade epochs was 30% higher than fixation epochs, it was subsequently flagged as an eye-artifact-related IC (Coco et al., 2020). Across all participants, on average 4.47 ICs (*SD* = 2.66) were marked for rejection. The impact of the ocular artifact removal on the data is visualized in Fig. 4, showing the grand averaged FRP at frontal and occipital electrodes with the raw data, corrected data, and isolated ocular ICs. Replicating previous work, the ocular ICs had the largest impact at frontal electrodes compared with occipital electrodes (Ries et al., 2016).

#### Linear deconvolution modeling

In addition to ocular artifacts, temporally adjacent ocular events (i.e., fixations and saccades), stimulus events, and behavioral events can overlap in time complicating the interpretation of neural activity in free-viewing paradigms. We used the Unfold Toolbox to alleviate this issue, which provides a deconvolution technique to calculate regression ERPs on the continuous EEG data (Smith & Kutas, 2015a) allowing for the correction of overlapping effects and accounting for nonlinear covariates (Dimigen & Ehinger, 2021; Ehinger & Dimigen, 2019). The 64-channel continuous EEG data from each participant was resampled to 256 Hz, re-referenced to the average of the left and right mastoids, and bad channels were detected using a voltage threshold of four standard deviations and spherically interpolated. Next, we transferred the resulting flagged unmixing weights from the OPTICAT processing to the copy of the EEG data for removal of ICs related to ocular artifacts. Finally, we filtered the EEG data using a second-order Butterworth bandpass filter from 0.1 Hz to 40 Hz. This continuous EEG data were used to define a design matrix for fixation events following the *unfold* analysis pipeline (Dimigen & Ehinger, 2021; Ehinger & Dimigen, 2019; Wunderlich & Gramann, 2020).

We defined two deconvolution models to evaluate the influence of low-level image statistics, one for amplitude spectrum slope and another for average luminance around fixation, on early visual processing while controlling for low-level covariates. Specifically, eye-movement factors, such as fixation duration, which can be responsible for the source overlapping effects from subsequent eye movements; saccade magnitude, which have a known effect on the lambda response; and saccade angle (Nikolaev et al., 2016; Ries et al., 2018). Since saccade magnitude has previously been shown to have a nonlinear relationship with the FRP (Nikolaev et al., 2016; Ries et al., 2018), this predictor can be modelled using a spline regressor (Dimigen & Ehinger, 2021; Ehinger & Dimigen, 2019; Smith & Kutas, 2015b). In each model, an additional factor called “fixation category” was included to account for additional differences between fixations occurring on objects in the scene compared with the background. Fixation category was based on raycast data (gaze ray collisions) from the virtual environment, resulting in two categories: object or scene background. Any fixation on placed objects (manmade objects) in the scene counted towards the object category, regardless of whether it was a target or nontarget, and any fixation not on an object (i.e., terrain, vegetation, sky) was considered scene background. For more information on this raycast process, see Enders et al. (2021). For each model, EEG activity for fixation onsets were modelled using predictors of saccade magnitude, saccade angle, and fixation duration as spline regressors with five splines. Average luminance, and slope of the amplitude spectra (using calculated α) were modelled as continuous factors, and fixation category, to account for remaining low-level image differences, as a categorical predictor.

Model 1 evaluates the impact of low-level image statics at each fixation, and the aim was to extend previous findings quantifying the influences of spatial frequency and luminance on the early evoked visual potentials (Ellemberg et al., 2001; B. C. Hansen et al., 2011, 2012; Ries & Hopfinger, 2011) and previous FRP findings from simplified tasks (Kazai & Yagi, 1999; Ossandón et al., 2010). In Model 1, we used the fixation onset events separated by a saccade that met the parameters outlined above in the Eye-Movement Data and Image Statistics sections. The Model 1 linear regression was defined as follows using the Wilkinson notation (Wilkinson & Rogers, 1973):

$${EEG}_{Fixation\, Onset}=1+spline\left(sacc.\, size,5\right)+spline\left(sacc.\, angle,5\right)+spline\left(fixation\, duration,5\right)+average\, luminance+amplitude\, spectra\, slope+cat\left(fixation\, category\right)$$

In Model 2, we wanted to understand how neural activity from temporally adjacent fixations were influenced by changes in the image statistics from one fixation to the next, and aimed to replicate previously observed changes in luminance and spatial frequency from consecutive fixations (Kazai & Yagi, 1999; Ossandón et al., 2010). In this model, we used consecutive fixation events separated by a saccade that met the parameters outlined above in the Eye-Movement Data and Image Statistics sections. In Model 2, we modelled the EEG activity of the second of the two fixations as the dependent variable and took the difference between the calculated predictor values between fixations, giving us a relative “contrast” between scene statistic values between fixation *n* − 1 and *n*. For ease of interpretation, we used the absolute value of the scene slope, or α, values. Negative predictor values represent that the first fixation had a larger predictor value than the second fixation. Positive predictor values represent that the second fixation had a larger predictor value than the first fixation. On average, a total of 741.30 consecutive fixations (*SD* = 311.86) per participant were included in Model 2:

$${EEG}_{2nd\, Fixation\, Onset}=1+spline\left(sacc.\, size,5\right)+spline\left(sacc.\, angle,5\right)+spline\left(fixation\, duration,5\right)+\Delta\, in\, average\, luminance+\Delta\, in\, amplitude\, spectra\, slope+cat\left(fixation\, category\right)$$

The linear deconvolution models followed the same *unfold* modeling steps.^1^ We *defined* the design matrix (function: *uf_designmat*) based on the above described models and *imputed* any missing values across predictors (function: *uf_imputeMissing*), and the design matrix was *time-expanded* 200 ms before and 400 ms after fixation onsets (function: *uf_timeexpandDesignmat*; Skukies & Ehinger, 2023). Next, we applied a *continuous artifact detection* set to amplitude threshold of 250 µV with a window size of 1,000 ms (function: *uf_continuousArtifactDetect*) and removed fixations during the math task interval (function: *uf_continuousArtifactExclude*). The linear models were then fit (function: *uf_glmfit)* and restructured (function: *uf_condense*). As part of postfitting in all models, we *predicted* saccade magnitude from 1° to 15° in increments of 1°. For Model 1 and 3, we *predicted* average luminance from 20 to 50 cd/m^2^ in increments of 10 and scene spatial frequency using α from − 0.9 to − 1.4 in increments of 0.1. For Model 2, we *predicted* luminance from − 20 to 20 cd/m^2^ in increments of 10 and *predicted* scene spatial frequency (α) at − 0.3 to 0.3 in increments of 0.1 (function: *uf_predictContinuous*). We used a period of 175 to 75 ms before fixation onsets for *baseline* correction. Finally, we added the marginals of other predictors (function: *uf_addmarginal*) and *extracted* the intercept and beta values for each predictor. The resulting betas from the deconvolution modeling can be interpreted as similar to traditional ERP waveforms, where the betas represent the amplitude estimated at a given time point (Smith & Kutas, 2015a). Once time-expanded, Model 1 had 47 predictors and Model 2 had 49 predictors resulting in a total of 2,448 columns. Across participants, the amount of model data varied because of the different number of events, but, on average, 268,009 rows (*SD* = 66,279) were used for the modeling.

#### Statistical measurements and analysis

The analysis focused on comparing the magnitude of the beta values, which represent amplitude of the regression FRP (rFRP) at an occipital region of interest (ROI: O1, Oz, O2; Ries et al., 2016) since the lambda response is typically maximum over electrode Oz (Billings, 1989; Yagi, 1981). The lambda response was defined as a positive deflection between 50 to 100 ms and the FRP N1 as negative deflection between 100 to 200 ms at the occipital ROI for each participant. We defined the peak amplitude for each component as the average of the beta values across two samples on either side (3.9 ms/sample) of the maximum within the time range for each FRP component, resulting in an average across five samples (or 19.5 ms). We used the average peak beta values for comparing component amplitudes between participants for group-level analysis focusing on overall changes in amplitude.

In addition to this main analysis, an additional control analysis to validate the continuous predictors findings was conducted and is presented in the supplementary materials (Appendix B). This provides an alternative approach to analyzing continuous predictors without using the “*uf_predictContinuous*” function. The result of this alternative analysis corroborates the repeated measures approach presented below.

The statistical analysis was conducted using the R software package (R Core Team, 2021). All frequentist repeated measures analyses of variance (ANOVAs) were conducted using the *ez* package (Lawrence, 2016) and Bayesian repeated-measures ANOVAs using the *BayesFactor* package (Morey & Rouder, 2018). A Greenhouse–Geisser correction for nonsphericity was used for ANOVAs where the corrected degrees of freedom and *p* values are provided along with the epsilon value. False discovery rate corrections were performed for follow-up *t* tests and corrected *p* values are provided (Benjamini & Hochberg, 1995). Effect sizes are provided for ANOVAs as generalized eta squared and Cohen’s *d* for *t* tests (Lakens, 2013). When appropriate, Bayesian statistics are given as BF_01_ or the amount of evidence in favor of the null model and are interpreted following Wagenmakers et al. (2018).

## Results

### Eye-movement characteristics

During the navigation task, participants made fixations that were on average 330.88 ms (*SD* = 48.84 ms) in duration and were accompanied by saccades that were on average 4.86° (*SD* = 0.66°) in magnitude. Since the virtual environment was composed of a mixture of a virtual terrain (e.g., terrain and sky) and virtual objects, we categorized each fixation into one of two categories—background or object fixations. Comparing eye-movement characteristics across the experiment and category, participants had on average more fixations on objects (*M* = 356.45, *SD* = 189.50) than the background (*M* = 271.79, *SD* = 118.09), *t*(32) = 2.78, *p* = 0.009, *d* = 0.52, and also looked longer at objects (*M* = 351.67 ms, *SD* = 59.09 ms), compared with the background (*M* = 302.87 ms, *SD* = 40.39 ms), *t*(32) = 9.43, *p* < 0.001, *d* = 0.78. Participant’s fixations on the background were accompanied by larger saccades (*M* = 5.12°, *SD* = 0.82°) compared with objects (*M* = 4.70°, *SD* = 0.66°), *t*(32) = 4.86, *p* < 0.001, *d* = 0.54. These results suggest there are underlying differences in the eye movement characteristics between the fixations categories, which will need to be handled as covariates in subsequent modeling.

### Observed average luminance and amplitude spectrum slopes

**Fig. 5:**
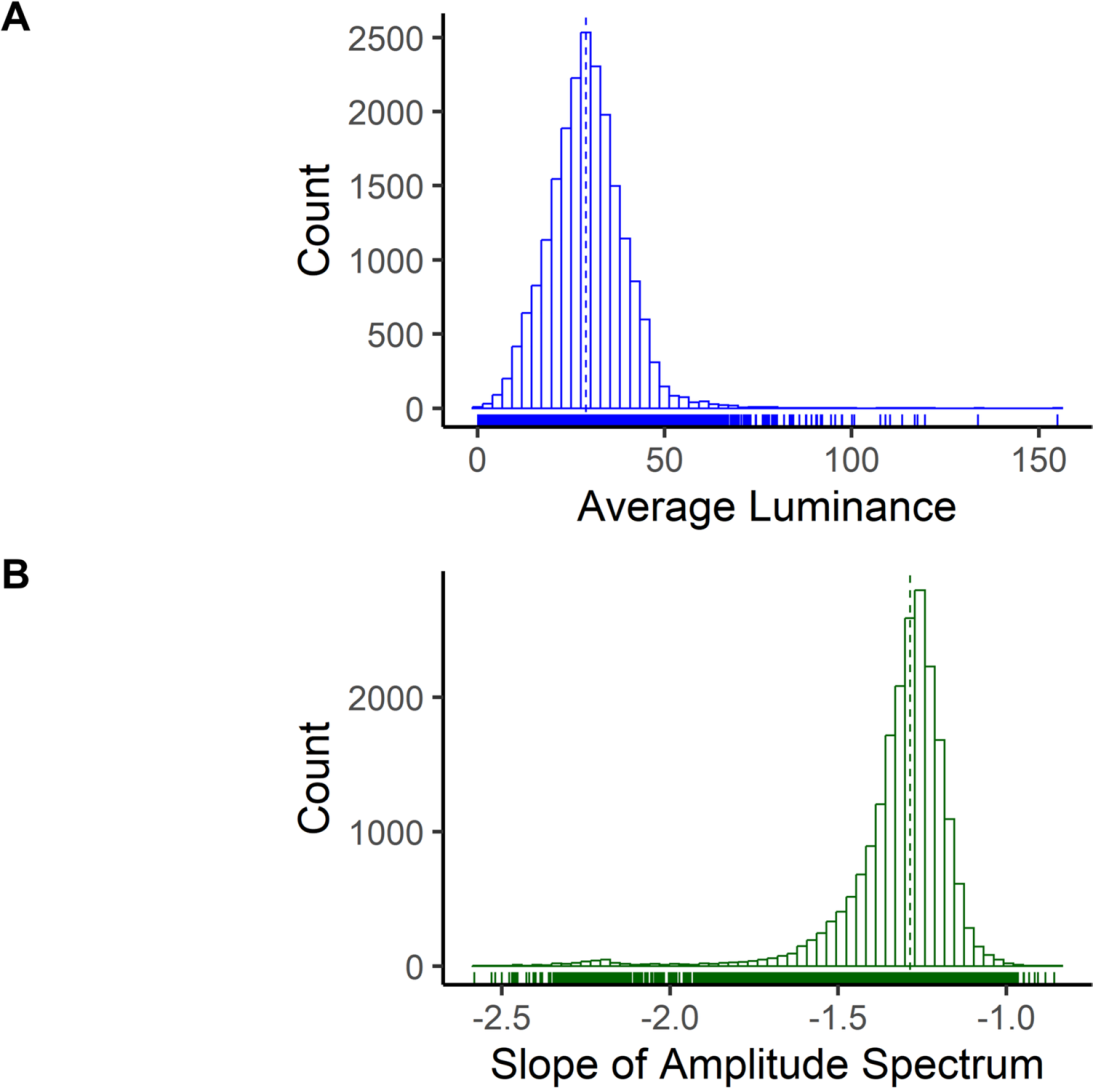
Observed average luminance and amplitude spectrum slope. *Note.* Distributions of all the participants’ observed average luminance and amplitude spectrum slopes at 5° patch around the fovea used in the deconvolution modeling analysis with the median plotted as a vertical dotted line

The distributions in Fig. 5 show the observed average luminance and amplitude spectrum slopes from all participants. Since we were unable to control how each participant experienced the virtual navigation task, which subsequently influenced the observed image statistics, we used these distributions to determine where all the participants overlapped in observed values and used them as prediction values. Across the experiment, participants’ mean average patch luminance was 29.49 cd/m^2^ (*SD* = 2.05 cd/m^2^), and the average amplitude spectrum slope was − 1.322 (*SD* = 0.06). Next, we compared the differences in image statistics across the two types of fixation categories of background versus object. Overall, the background had a higher average luminance (*M* = 32.00 cd/m^2^, *SD* = 2.22 cd/m^2^) compared with objects (*M* = 27.48 cd/m^2^, *SD* = 1.97 cd/m^2^), *t*(32) = 12.98, *p* < 0.001, *d* = 2.15. There was also a difference in observed amplitude spectrum slopes, with background objects having a steeper slope (*M* = − 1.33, *SD* = 0.063) compared with object fixations (*M* = − 1.31, *SD* = 0.060), *t*(32) = 2.91, *p* = 0.0065, *d* = 0.29. These results suggest there are underlying differences in the low-level observed image statistics between the fixations categories, supporting the inclusion in the modeling to account of other potential low-level visual features.

### Low-level image statistics influence on the fixation related potential (Model 1)

**Fig. 6:**
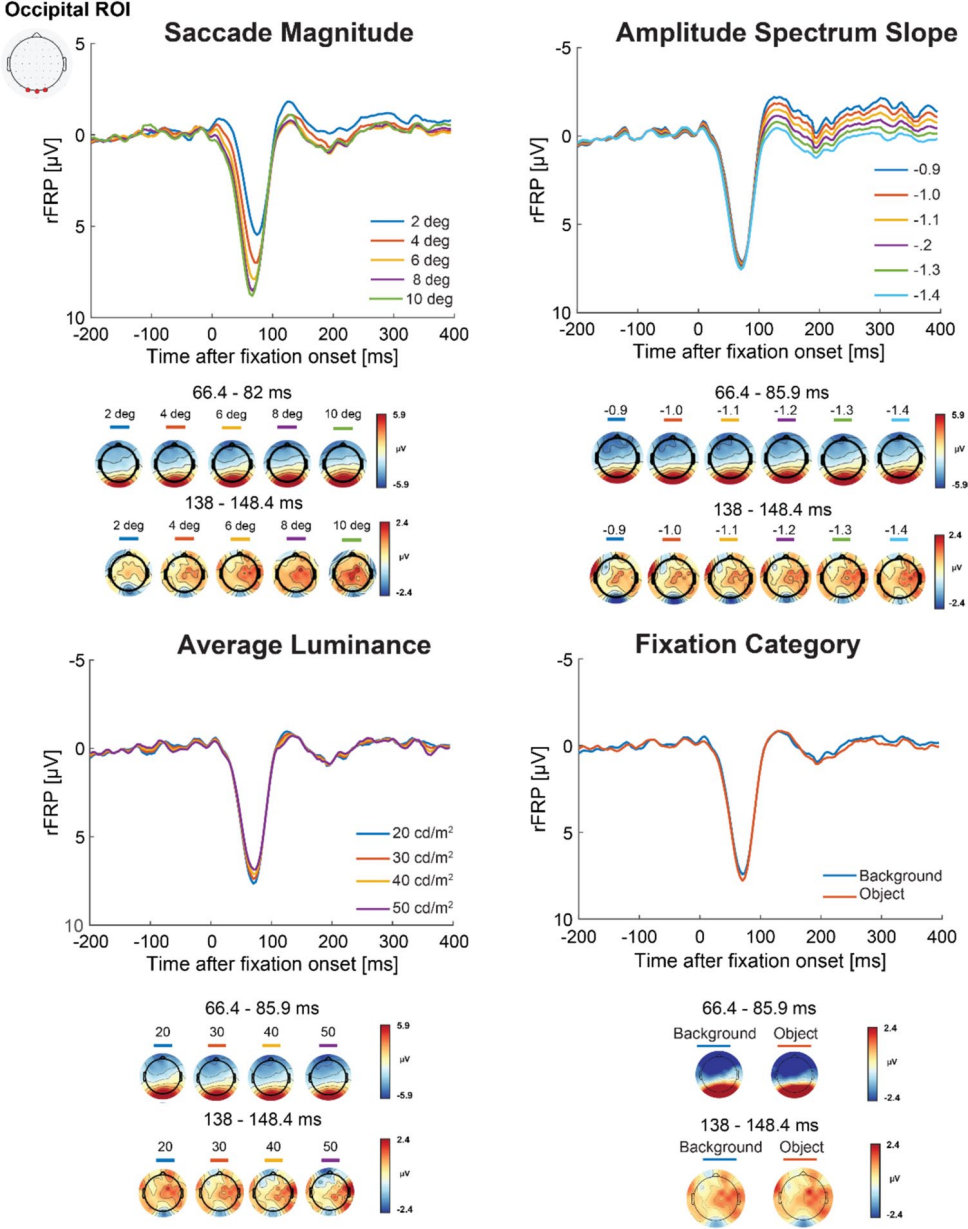
Model 1 grand averaged rFRP and topographical maps. *Note.* The Model 1 grand average rFRPs (in microvolts) for each predictor with positive plotted down at the occipital ROI shown with the corresponding topographical maps averaged across all electrodes for the peak latency for each predictor. (Color figure online)

**Fig. 7:**
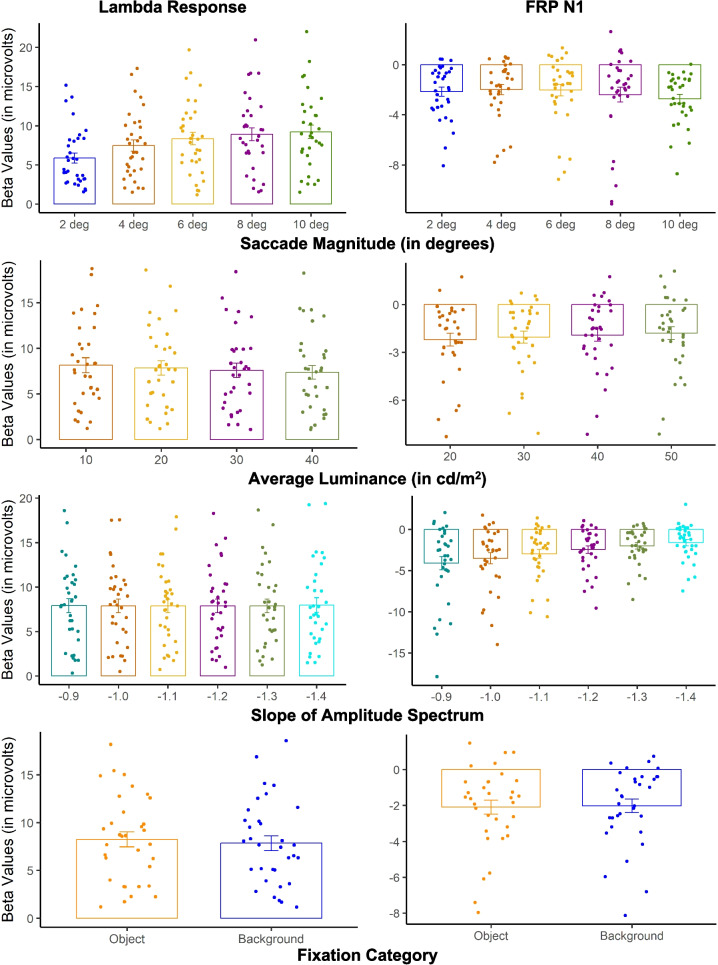
Model 1 beta values for the occipital ROI. *Note.* The betas values for each Model 1 predictor by FRP component with subject-level variability shown as individual data points and group-level means represented by bar plots with the error bars as the between-subject standard error. (Color figure online)

The aim of Model 1 is to evaluate the influence of image statistics and semantic information on the FRP while controlling for the influence of saccade size, saccade angle, and fixation duration. Figure 6 depicts the grand average rFRP for each model predictor at an occipital ROIs with the corresponding topographic maps for all scalp electrodes during the mean peak latency across the predictor values. Figure 7 shows the Model 1 beta values for each predictor value representing the amplitude for each FRP component.

#### Saccade magnitude

Due to the nonlinear effect of saccade size on the FRP (Nikolaev et al., 2016; Ries et al., 2018), we analyzed the lambda response and FRP N1 amplitudes for even saccade sizes up to ten degrees. At the occipital ROI, we found a prominent positive deflection occurring on average 74.20 ms from fixation onset across all conditions corresponding to the lambda response (see Fig. 6). Using a one-way repeated-measures ANOVA, there was significant main effect of saccade magnitude on the lambda response amplitude, *F*(1.57,50.14) = 52.81, *p* < 0.001, ε = 0.39, η_*G*_^2^ = 0.07. Follow-up paired *t* tests confirmed that larger saccades lead to larger lambda response amplitudes at the occipital ROI. The lambda response amplitude significantly increased from two to four degrees, *t*(32) = 7.44, *p* < 0.001, *d* = 0.36, from four to six degrees, *t*(32) = 7.49, *p* < 0.001, *d* = 0.17, and from six to eight degrees, *t*(32) = 4.67, *p* < 0.001, *d* = 0.12. Following the previously found nonlinear increases in lambda response amplitude and saccade size (Nikolaev et al., 2016; Ries et al., 2018), the difference between 8° and 10° saccades was not significant, *t*(32) = 2.05, *p* = 0.050, *d* = 0.06. A Bayesian paired *t* test provided no evidence of a difference (BF_01_ = 0.85 ± 0.02%).

Occurring on average 141.00 ms from fixation onset across all saccade size predictor values, we found a negative deflection corresponding to the FRP N1 at the occipital ROI. There was, however, no effect of saccade magnitude on the FRP N1, *F*(1.52,48.71) = 1.97, *p* = 0.16, ε = 0.38, η_*G*_^2^ < 0.01. A follow-up Bayesian repeated-measures ANOVA provided strong evidence in support of no difference between saccade size on the FRP N1 amplitude (BF_01_: 2.85 ± 0.54%).

#### Average luminance

Next, we evaluated the effect of average luminance within a 5° patch around fixation on the lambda response and FRP N1 amplitudes**.** At the occipital ROI, we found a prominent positive deflection in the FRP occurring on average 75.99 ms from fixation onset across all luminance predictor values (shown in Fig. 6). Using a one-way repeated-measures ANOVA, we found a significant main effect of luminance on the lambda response amplitude, *F*(1.01,32.32) = 5.19, *p* = 0.029, ε = 0.34, η_*G*_^2^ < 0.01. Follow-up paired *t* tests confirmed significant differences the luminance predictor values of 20 and 30 cd/m^2^, *t*(32) = 1.97, *p* = 0.036, *d* = 0.05, where lower luminance resulted in a larger lambda response amplitude. The difference in luminance predictors 30 to 40 and 40 to 50 cd/m^2^, however, were not significant (all *t* values < 2.16, *p* values = 0.057, *d* = 0.050–0.058).

We found a negative deflection corresponding to the FRP N1 at the Occipital ROI occurring on average 137.78 ms from fixation onset across all luminance predictor values. A one-way repeated-measures ANOVA using the FRP N1 amplitude for luminance predictor suggested there was no significant difference, *F*(1.14,36.56) = 2.34, *p* = 0.132, ε = 0.38, η_*G*_^2^ < 0.01. A follow-up Bayesian repeated-measures ANOVA provided strong evidence in support of no influence of luminance on the FRP N1 amplitude (BF_01_: 1.75 ± 1.32%).

#### Amplitude spectrum slopes

Then we evaluated the influence of the amplitude spectrum slope around a 5° patch at fixation on the lambda response and FRP N1 amplitude**.** For this predictor, we used the computed α values representing the slope of the amplitude spectrum. Shown at the bottom of Fig. 5, at the occipital ROI, a prominent positive deflection corresponding to the lambda response was found on average 75.99 ms from fixation onset across α predictor values. A one-way repeated-measures ANOVA using lambda response amplitude for the α predictor values showed there was no significant difference between any of the α predictor values on the lambda response amplitude, *F*(1.01,32.36) = 0.03, *p* = 0.86, ε = 0.20, η_*G*_^2^ < 0.01. Consistent with this finding, a Bayesian repeated-measures ANOVA suggested there was strong evidence of no influence of scene spatial frequency on the lambda response (BF_01_ = 91.74 ± 0.45%).

Consistent with the other model predictors, for the slope of the amplitude spectrum we found prominent negative deflection occurring on average 135.20 ms from fixation onset at the occipital ROI across all α values corresponding to the FRP N1. We found a significant influence of the α predictor values on the FRP N1 amplitude using a one-way repeated-measures ANOVA, *F*(1.01,32.28) = 10.70, *p* = 0.003 ε = 0.20, η_*G*_^2^ = 0.07. Follow-up paired *t* tests confirmed significant differences between each α predictor value where smaller scene slope values (e.g., − 0.9) corresponding to greater contrast energy at higher spatial frequencies resulted in a larger N1 amplitude (all *t* values > 2.72, *p* values ≤ 0.010, *d* = 0.076–0.19).

#### Fixation categories

Finally, we evaluated the fixation categories to capture semantic information at fixation and other potential for low-level image differences that may exist as a categorical variable. Shown in Fig. 6, we found a prominent deflection in the FRP at the occipital ROI on average 76.35 ms from fixation onset consistent with the lambda response. Using a paired *t* test on the lambda response amplitudes, there was a significant difference between fixations on objects or the background, *t*(32) = 2.43, *p* = 0.02, d = 0.09, where object fixations led to a larger lambda response amplitude. Next, we compared the FRP N1 amplitudes, which were a negative deflection occurring on average 135.42 ms from fixation onset at the occipital ROI. A paired *t* test between fixation categories on the FRP N1 amplitudes showed no significant difference, *t*(32) = 0.50, *p* = 0.62, *d* = 0.03. A follow-up Bayesian *t* test suggests there is strong evidence of no influence of fixation category on the N1 (BF_01_ = 4.78 ± 0.04%).

### Validation of deconvolution modeling

A third model, Model 3, is presented in the supplementary materials (Appendix A) that validates the overall modeling approach by randomly shuffling the observed low-level image statistics predictor values associated with different eye-movement events and neural activity to evaluate the impact of randomly assigned values. Here, the effects of observed low-level image statistics should disappear since they are no longer yoked to the actual eye-movement events and neural activity. As expected, this randomization resulted in no effect of the low-level image statistics on the FRP.

### Influence of image statistics on FRP between consecutive fixations (Model 2)

**Fig. 8:**
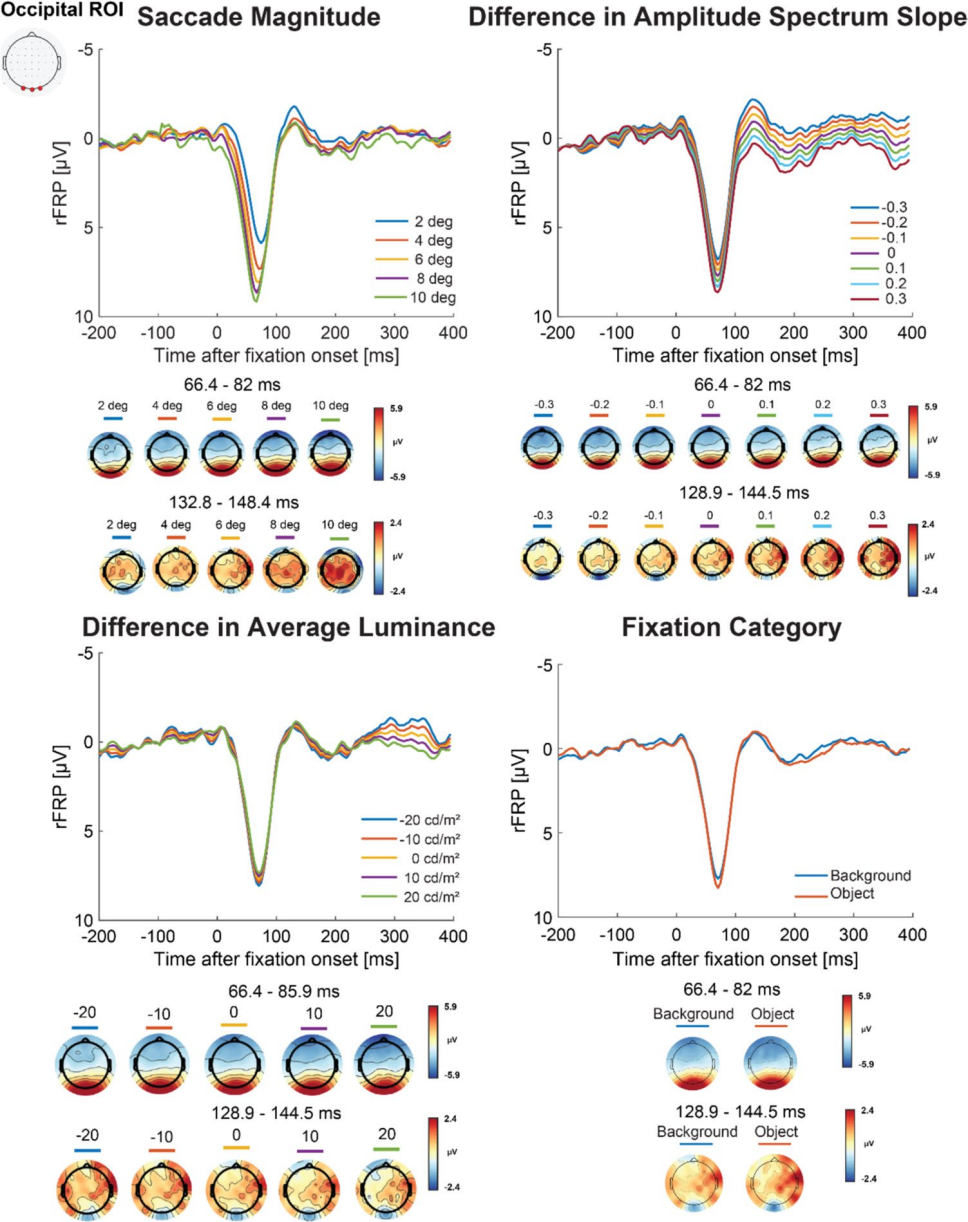
Model 2 Grand averaged rFRP with corresponding topographical maps. *Note.* The Model 2 grand average rFRPs (in microvolts) for each predictor, with positive plotted down at the occipital ROI shown with the corresponding topographical maps averaged across all electrodes for the peak latency across for each predictor. (Color figure online)

**Fig. 9:**
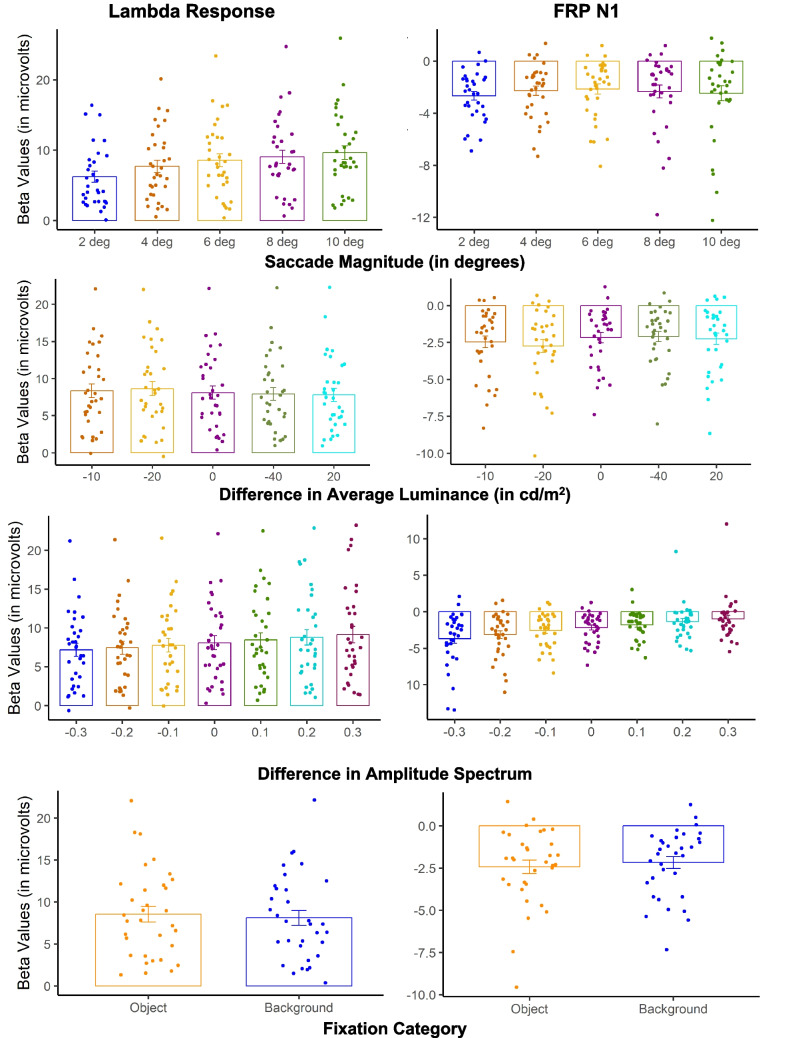
Model 2 beta values for occipital ROI. *Note.* This figure shows the betas values for each predictor in Model 2, with the subject-level variability as individual data points and group-level means represented by bar plots with the error bars as the between-subject standard error. (Color figure online)

During scene perception, natural visual behaviors often involve multiple fixations. Following an idea from Kazai and Yagi (1999), the observed FRP might be influenced from activity across temporally adjacent fixations. The aim of Model 2 was to investigate how changes in low-level image statistics from temporally adjacent fixations influence neural activity. In Model 2, we modelled the neural activity of the onset of the second fixation from two temporally adjacent fixations. For the model predictors, we used the difference between the average luminance and slope of the amplitude spectrum between the two temporally adjacent fixations. Specifically, we used the predictor values used in Model 1 and computed the change for each image statistic value where we subtracted the value from the first fixation (*n* − 1) from the value from the second fixation (*n*). Thus, in Model 2, negative predictor values indicate that the first fixation had a larger predictor value than the second fixation, and positive predictor values represent that second fixation had a larger predictor value than the first fixation. Figure 8 shows the grand average rFRP for each predictor from Model 2 at the occipital ROI with corresponding topographical maps for all scalp electrodes during the mean peak latency across the predictor values. Figure 9 shows the Model 2 beta values representing the amplitude for each predictor value and FRP component.

#### Saccade size

Consistent with the lambda response, at the occipital ROI we found a positive deflection on average 73.55 ms from fixation onset across the saccade magnitude predictors (shown in Fig. 8). We used a one-way repeated-measures ANOVA on the lambda response amplitudes to evaluate the influence of saccade size, which showed a significant effect, *F*(1.63,52.21) = 39.86, *p* < 0.001, ε = 0.41, η_*G*_^2^ = 0.05. Follow-up paired *t* tests showed that as saccade size increased so did the lambda response amplitude (all *t* values ≥ 3.26, *p* values ≤ 0.0025, *d* = 0.09–0.30). Consistent with the FRP N1, at the occipital ROI we found a prominent negative deflection on average 141.71 ms from fixation onset across saccade magnitudes. We used a one-way repeated-measures ANOVA on the FRP N1 amplitudes to evaluate the influence of saccade size, which was not significant, *F*(1.55,49.70) = 0.63, *p* = 0.497, ε = 0.39, η_*G*_^2^ = 0.01. A Bayesian repeated-measures ANOVA showed strong evidence of no difference of the FRP N1 amplitude by saccade magnitude (BF_01_: 20.40 ± 0.49%).

#### Difference in average luminance

Across the difference in luminance predictor values at the occipital ROI, we found a prominent positive deflection on average 73.63 ms from fixation onset that is consistent with the lambda response (shown in Fig. 8). We used a one-way repeated-measures ANOVA on the lambda response amplitudes for each luminance difference predictor value to evaluate the influence of changes in average luminance which showed a nonsignificant effect, *F*(1.03,32.96) = 4.20, *p* = 0.047, ε = 0.26, η_*G*_^2^ < 0.01. Follow-up paired *t* tests indicated the only significant difference was between the predictor values of − 20 and − 10 cd/m^2^, *t*(32) = 2.93, *p* = 0.0025, *d* = 0.061, but not between − 10 and 0 cd/m^2^ (or no difference in luminance across fixations), *t*(32) = 2.33 *p* = 0.051, *d* = 0.045, 0 and 10 cd/m^2^, *t*(32) = 1.72, *p* = 0.13, *d* = 0.095, or 10 and 20 cd/m^2^, *t*(32) = 1.13, *p* = 0.26, *d* = 0.028. Overall, the pattern of lambda response amplitudes was consistent with Model 1. That is, lower luminance on the second fixation led to a larger lambda response amplitude (e.g., luminance difference equal to − 20 cd/m^2^ is where the first fixation, *n* − 1, had an average patch luminance of 30 cd/m^2^ and second fixation, *n*, had an average patch luminance of 10 cd/m^2^).

We found a negative deflection in the FRP at the occipital ROI occurring on average 135.58 ms from fixation onset across the difference in luminance predictor values consistent with the FRP N1. We first used a one-way repeated-measures ANOVA on the FRP N1 amplitudes to evaluate the influence of differences in average luminance, which no significant effect, *F*(1.26, 40.39) = 2.22, *p* = 0.138, ε = 0.32, η_*G*_^2^ = 0.01. A Bayesian repeated-measures ANOVA provided no evidence, however, of similar FRP N1 amplitudes across the differences in luminance between fixations (BF_01_: 0.11 ± 0.42%).

#### Difference in spatial frequency

Shown in Fig. 8, we found a prominent positive deflection in the FRP on average 74.29 ms from fixation onset consistent with the lambda response across all α predictor values at the occipital ROI. To calculate the differences between spatial frequency on consecutive fixations and for ease of interpretability, we used the magnitude of the slope or the absolute value of α. In this way, smaller slopes (i.e., − 0.9 became 0.9; associated with greater contrast at higher spatial frequencies) subtracted from larger slopes (i.e., − 1.4 became 1.4; associated with greater contrast at lower spatial frequencies) would result in a negative difference value representing that there was a higher spatial frequency on the first fixation (*n*) compared with the second fixation (*n* − 1). Conversely, a positive value indicated that there was a higher absolute slope value, (i.e., 1.4) on the *n* fixation compared with the *n* − 1 fixation (i.e., 0.9), which would result in a positive difference value representing that there was a decrease in spatial frequency. Overall, the lambda response follows a similar pattern found in Model 1, where a positive difference in α, associated with a lower spatial frequency on the second fixation than the first fixation, resulted in a larger lambda response amplitude. To test this effect, we first did a one-way repeated-measures ANOVA on the lambda response amplitudes (shown on the left side of Fig. 8) which showed a significant effect, *F*(1.01,32.38) = 7.91, *p* = 0.008, ε = 0.17, η_*G*_^2^ = 0.02. All follow up testing between the differences in scene spatial frequency were significant (*t* values ≥ 2.48, *p* values ≤ 0.018, *d* = 0.055–0.065).

We found a negative deflection at the occipital ROI occurring on average 135.97 ms from fixation onset across all spatial frequency predictor values, which is consistent with the timeframe of the FRP N1. As before, the pattern of the amplitudes reversed from the lambda response to the FRP N1, where greater contrast at high spatial frequencies at the second fixation led to a larger FRP N1 amplitude. To test this effect, we first did a one-way repeated-measures ANOVA on the FRP N1 amplitudes which showed a significant difference between spatial frequency predictor values, *F*(1.02,32.58) = 8.23, *p* = 0.007, ε = 0.17, η_*G*_^2^ = 0.10. All paired *t* tests between the differences in spatial frequency were significant (*t* values ≥ 2.40, *p* values ≤ 0.022, *d* = 0.11–0.19).

#### Fixation categories

Finally, we compared the FRP activity related to fixation categories that allowed us to account for potential differences in low-level visual features not captured by the luminance and amplitude spectrum slope predictors. We found a prominent deflection in the FRP at the occipital ROI on average 73.75 ms from fixation onset consistent with the lambda response. Using a paired *t* test on the lambda response amplitudes, there was a significant difference between fixations on objects versus background, *t*(32) = 02.85, *p* = 0.0075, *d* = 0.085, where object fixations resulted in larger lambda response amplitudes. Next, we compared the FRP N1 amplitudes, which were a negative deflection occurring on average 134.47 ms from fixation onset at the occipital ROI. A paired *t* test between fixation categories on the FRP N1 amplitudes showed no significant difference of fixation category on the FRP N1 amplitude, *t*(32) = 1.39, *p* = 0.174, *d* = 0.11. A follow up paired Bayesian *t* test suggested there was evidence of no difference between the groups (BF_01_: 2.23 ± 0.03%).

## Discussion

Previous work on electrophysiological correlates of scene perception have primarily used traditional EEG experimental setups that limit eye movements to minimize ocular artifacts. In doing so, these paradigms inadvertently create situations where participants passively view scenes instead of playing an active role in the selection of visual information via eye movements. In this study, we used an approach that combines co-recording of eye tracking and EEG with recent state-of-the-art signal-processing techniques to study FRPs related to scene perception. Specifically, we used a signal-processing method that allowed us to optimize the removal of eye-movement artifacts using OPTICAT (Dimigen, 2020) and to control for covariates of the FRP (e.g., saccade magnitude, saccade angle, fixation duration) while overcoming issues from overlapping neural activity through deconvolution modeling (Dimigen & Ehinger, 2021; Ehinger & Dimigen, 2019). By combining the FRP approach with deconvolution modeling, this work demonstrates that FRP components can be disentangled from aspects that are influenced by eye movements from those that could track or measure visual and cognitive processing. We used a navigation and visual search task in a virtual environment presented on a computer screen, which allowed us to compute post hoc image statistics of luminance and spatial frequency to evaluate the influence on the FRP. To our knowledge, this is one of the first experiments using a complex, dynamic, continuous scene to demonstrate the lambda response and fixation-related N1 are independently modulated by image statistics such as luminance and measures of spatial frequency while controlling for the influence of eye-movement characteristics.

### Influence of image statistics on the FRP

Model 1 evaluated the influence of low-level image statistics on neural activity associated with fixation onsets while controlling for the effect of saccade magnitude, saccade angle, and fixation duration (Dandekar et al., 2012; Dimigen et al., 2011; Nikolaev et al., 2016; Ries et al., 2018; Thickbroom et al., 1991). The results suggest early FRP components are sensitive to these low-level visual features independently from saccade magnitude. Specifically, the results from Model 1 found a lower luminance at fixation resulted in a larger lambda response when controlling for local spatial frequency and eye-movement characteristics. The effect of scene spatial frequency on the fixation-related N1 was significant, showing that greater contrast at higher spatial frequencies resulted in a larger N1 amplitude when controlling for luminance and saccade magnitude.

Model 2 evaluated the influence of changes in image statistics across consecutive fixations on the FRP consistent with the idea that neural activity from one fixation is influenced by activity from the prior fixation (Kazai & Yagi, 1999). As before, we found when there was an extreme change from a lower luminance on the second fixation a larger lambda response was observed. Interestingly, in Model 2 the effect of scene spatial frequency had a significant influence on the amplitude of the lambda response and fixation-related N1, where greater contrast at lower spatial frequencies on the second fixation resulted in a larger lambda response amplitude and greater contrast at higher spatial frequencies on the second fixation resulted in a larger fixation-related N1 amplitude. Together, Models 1 and 2 suggest that image statistics observed around fixation independently influence the FRP, while controlling for ocular artifacts, low-level influences, and overlapping activity from temporally adjacent events.

Contrary to prior work on the influence of luminance using ERPs, which showed larger luminance typically result in larger amplitudes of early visually evoked components (Johannes et al., 1995) and from a previous FRP study showing that larger absolute changes in luminance yield larger P1 amplitudes (Ossandón et al., 2010), we found the opposite effect of luminance on the lambda response. The effect of luminance observed in our deconvolution modeling was that lower luminance led to larger lambda response. The counterintuitive finding might be the result of the dynamic or continuous stimulus, where in other previous studies the stimuli were static or changed between trials across discrete intervals. Another possibility is that the larger FRP amplitude with lower luminance is the result of a divisive normalization process (Carandini & Heeger, 2012). Divisive normalization adjusts the responses of neurons by normalizing the response of a given stimulus by considering the sum of the responses from neighboring neurons. It then follows that as a stimulus luminance increases, the response of the early visual cortex neurons will be reduced relative to the response of other neurons in the area, thus resulting in lower amplitudes from early visually evoked potentials. Thus, the opposite holds that as luminance is lower, such as at a 5° patch from fixation, from other areas of visual cortex that are stimulated, a higher neuronal response might occur resulting in a higher amplitude from early visually evoked potentials. To determine if this was a possibility, we compared the overall luminance from the full image of the virtual environment to the luminance at the 5° patch, which showed that indeed the full image of virtual world was on average brighter than the 5° patch. As a result, lower luminance patches would be farther away from the mean luminance of the full image than the higher luminance patches, making divisive normalization a potential mechanism for the observed larger amplitudes at lower observed luminance predictor values.

Overall, the effect of low spatial frequency on the lambda response followed by the effect of high spatial frequency on the N1 is consistent with coarse-to-fine processing (Groen et al., 2013; Hegde, 2008; Kauffmann et al., 2014, 2015; Petras et al., 2019) and previous ERP studies (Ellemberg et al., 2001; B. C. Hansen et al., 2011, 2012; Ries & Hopfinger, 2011). The effect of spatial frequency on the lambda response amplitude was not significant in Model 1, but in Model 2 when looking at changes across the consecutive fixations. One potential reason we did not observe a large effect of lower spatial frequency on the lambda response in Model 1 was that prior work using simple Gabor stimuli had a lower range of lower spatial frequencies (Ries et al., 2018) than what was typically observed in this experiment using a complex virtual environment. That is, if amplitude spectrum slopes were calculated on similar Gabor patches, they would have slope values that are on the steeper end of the overall observed values in the study. Another possibility is that since the difference between Models 1 and 2 were the modelled fixation events, where in Model 1 any fixation meeting the eye-movement parameters were included, whereas in Model 2 only consecutive or temporally adjacent fixations were included, the difference between the models and findings might suggest that the FRP is influenced by activity not just from the current fixation but from the fixation prior (Kazai & Yagi, 1999). Indeed, prior work by Kazai and Yagi (1999) using checkerboards looked at the influence of changes across consecutive fixations and with varying pattern sizes. They included a condition where across two fixations the checkboard pattern reversed from small (or high spatial frequency) to large (or low spatial frequency) pattern sizes, and they found that the lambda response amplitude was larger compared with when the checkerboard pattern stayed the same or went from large to small, whereas the N1 was the opposite and had a larger N1 amplitude when the pattern changed from a large to smaller checker pattern. Thus, in Model 1, our findings might have been diluted due to fixations that were not consecutive with each other, so the change in spatial frequency from one fixation to the next or influence from a prior fixation could not be properly modelled.

Including a categorical predictor to capture aspects of the scene at fixation (i.e., object versus background fixations) in both Models 1 and 2 allowed for accounting for other underlying image differences not captured by other predictors (Nikolaev et al., 2023b). In both models, fixations on objects resulted in larger lambda response compared with fixations on the background. Based on prior ERP research, this lambda response modulation may result from systematic differences between these stimuli, such as the relative distribution of edges and contours between objects and terrain (B. C. Hansen et al., 2011).

### Limitations and future directions

The results from this study are a necessary step to move from high experimenter-controlled contexts, such as fixed viewing conditions, to more ecological validity contexts the include eye movements allowing us to study visual behavior in more naturalistic situations, such as immersive virtual reality environments, or even real-world contexts. A limitation to this work is that we used a stimulated virtual environment presented on a computer screen. Although this provided a more complex, dynamic stimulus compared with previous FRP studies and the ability to calculate post hoc image statistics, due to the use of modelled objects and a generated artificial terrain, limitations in range of luminance range might have been present in scene. Additionally, differences could arise from this study and studies using more immersive (e.g., virtual reality) or real-world scenes due to the difference in the field of view of the stimulus leading to potential differences with stimulation in peripheral vision.

While the work presented in this paper takes a step towards understanding neural activity arising from naturalistic eye-movement behavior during a navigation task, it sparks many new research questions. The focus of this paper was to evaluate differences related to the early neural evoked activity, but there are many potential interesting differences occurring at later timeframes that were beyond the scope of this study. For example, there appears to be systematic differences between the slope of the amplitude spectrum predictors in the later temporal periods of the FRP (i.e., 200–400 ms). However, this only occurs at the Occipital ROI (comparing against central and parietal electrodes). Future work should explore the temporal differences of the influence of low-level image statistics on the FRP. Future work should also extend the findings from this study using a more realistic stimulus that has a higher dynamic range and provides a larger field of view, perhaps using virtual reality and photorealistic photospheres or 360-degree photos. Although not the primary focus here, future experiments should be designed to follow up on the results from Model 2 that explore how neural activity from consecutive fixations can influence the FRP and as well as more systematic evaluations of object processing using FRPs (see Coco et al., 2020). Direct applications of this work would translate well to work related to the parafoveal preview in reading (Antúnez et al., 2022; Mirault et al., 2020) or from look ahead effect in foraging visual search tasks (Kosovicheva et al., 2020).

### Conclusion

Every day we navigate environments that fluctuate in low-level visual features based on our position in space and different lighting conditions. In turn, the low-level visual information directly influences the neural response as we move our eyes around our environment and dynamically change our position in it. Co-recording of eye tracking and EEG to create FRPs with a dynamic, complex stimulus allows us to study visual processing in a naturalistic setting and investigate how low-level visual features, such as luminance and spatial frequency, influence neural activity with naturally occurring eye behaviors. Studying visual behavior and corresponding neural activity in this naturalistic setting provides a functional perspective of how visual processing occurs in the real world.

## Supplementary Information

Below is the link to the electronic supplementary material.

Supplementary file1 (DOCX 431 KB)

## Data Availability

The data underlying our findings, which can be used to recreate figures, tables, and statistical analysis in the current study, are available at a project on the URL listed below (https://osf.io/tex2w/?view_only=186e39e433eb417eb9aae4d08e0079d7). However, the raw datasets are only available upon reasonable request, as data analysis on other aspects of the data are still underway. In order to share raw data from all subjects, we must undergo an operational security check per Army Regulations 380–5 and AR-530–1 in order for them to be uploaded to a public repository. Nevertheless, we will perform all the necessary steps to meet the Army Regulations for all interested researchers wishing to obtain the data upon reasonable request to the corresponding author.

## Footnotes

1 Processing steps in the Unfold Toolbox are included as italicized font to indicate specific steps.

## Declarations

The views and conclusions contained in this document are those of the authors and should not be interpreted as representing the official policies, either expressed or implied, of the Army Research Laboratory or the U.S. Government. The U.S. Government is authorized to reproduce and distribute reprints for Government purposes notwithstanding any copyright notation herein.

### Open practices

The data underlying our findings, which can be used to recreate figures, tables, and statistical analysis in the current study, are available (https://osf.io/tex2w/?view_only=186e39e433eb417eb9aae4d08e0079d7). The materials for the current experiment are not available, and this study was not preregistered.
