# Supplementary material for: Fixation-related potentials during a virtual navigation task: The influence of image statistics on early cortical processing
Source: Atten Percept Psychophys. 2025 Jan 23;87(1):261–83. doi: 10.3758/s13414-024-03002-5 (PMC11845547; doi:10.3758/s13414-024-03002-5)
Supplement: Supplementary file 1 — Supplementary file1 (DOCX 431 KB) [file 13414_2024_3002_MOESM1_ESM.docx]

**Supplementary Material**

**Appendix A: Randomized Image Statistic Predictors**

In an approach to validate the deconvolution modeling approach, we ran an additional model, Model 3, where the low-level image statistic predictors from Model 1, both average luminance and amplitude spectrum slopes, were randomly shuffled and assigned to fixation events and the EEG activity was modelled with eye movement covariates predictors (e.g., fixation duration, saccade size, and saccade angle). The aim of Mo­del 3 is to validate the deconvolution modeling approach by randomizing the computed image statistics associated with fixation events, rerunning the modeling, and evaluating the effects to determine if they disappear when the image statistics are no longer yoked to the actual eye events and subsequent EEG activity. The image statistics values were randomly shuffled, conservatively, by using a random number seed and assigning the observed image statistics values to fixation events randomly. This approach means that some of the image statistic values could be assigned to the same fixation event due to random chance. In essence, this model was the same as Model 1, but included randomly shuffled image statistics predictor values. The predictor values for saccade magnitude were not randomly shuffled, so the following analysis focuses on the influence of low-level image properties on the FRP. This resulted in the following model:

$$\text{EEG }_{\text{Fixation Onset}}\text{ = 1+ spline(sacc. size, 5) + spline(sacc. angle, 5) + spline(fixation duration}\text{, 5) }\text{}$$

$\text{+ randomized average luminance + randomized amplitude spectra slope}$ + cat(fixation category)

Overall, we did not find significant differences on either the lambda response or N1 FRPs for either luminance or spatial frequency predictors (see Figures A1 and A2). This finding suggests that the deconvolution modeling was successful in relating scene statistic predictors to neural activity after fixation events. Figure A1 depicts the grand average rFRP from an occipital ROI for each randomized predictor, with the corresponding topographic maps for all the scalp electrodes during the mean peak latency for each predictor value. Figure A2 shows the subject-level variability and group-level means of the peak amplitude for each FRP component at each predictor value in Model 3.

***Randomized Luminance***

Shown on the top of Figure A1, we found a prominent positive deflection at 75.58 ms that corresponds to the lambda response. We used a repeated measures one-way ANOVA to compare the lambda response amplitudes for the randomized luminance predictor values. The results suggested there was no significant difference, F(1.08, 34.49) = 1.77 , *p* = 0.193, ε = 0.36 , 𝜂_𝐺_^2^ < 0.01, and confirmed with a Bayesian repeated measures ANOVA showing moderate evidence of no difference (BF_01_ = 3.36 ± 0.5%). Corresponding to the FRP N1, we found a negative deflection around 139.56 ms. Using the N1 amplitudes from the randomized luminance predictor, a one-way repeated measures ANOVA also showed no difference F(1.03, 33.07) = 0.79 , *p* = 0.384, ε = 0.34 , 𝜂_𝐺_^2^ < 0.01 that consistent with a Bayesian repeated measures ANOVA showing strong evidence of no difference (BF_01_ = 10.10 ± 0.75%).

***Randomized Spatial Frequency***

Shown on the bottom of Figure A1, a prominent positive deflection at 74.14 ms consistent with the lambda response was found. A repeated measures one-way ANOVA on the lambda response amplitude from the randomized α predictor values, representing scene spatial frequency, suggested there was no significant difference between conditions F(1.13, 36.26) = 2.49, *p* = 0.12, ε = 0.23 , 𝜂_𝐺_^2^ < 0.01. However, a follow-up Bayesian repeated measures ANOVA on the lambda response amplitudes from the randomized α predictor suggested anecdotal evidence of no difference (BF_01_ = 1.16 ± 0.41%). A negative deflection at 137.69 ms consistent with the FRP N1 was found. Using the N1 amplitudes from the randomized α predictor suggested there was no difference between conditions F(1.07, 34.14) = 0.95 , *p* = 0.342, ε = 0.21 , 𝜂_𝐺_^2^ < 0.01. This was corroborated by a repeated measures Bayesian ANOVA on the N1 amplitude, which suggested strong evidence of no difference (BF_01_ = 18.23 ± 0.56%).

**Figure A1**

*Model 3 Grand averaged rFRP with Corresponding Topographical Maps*
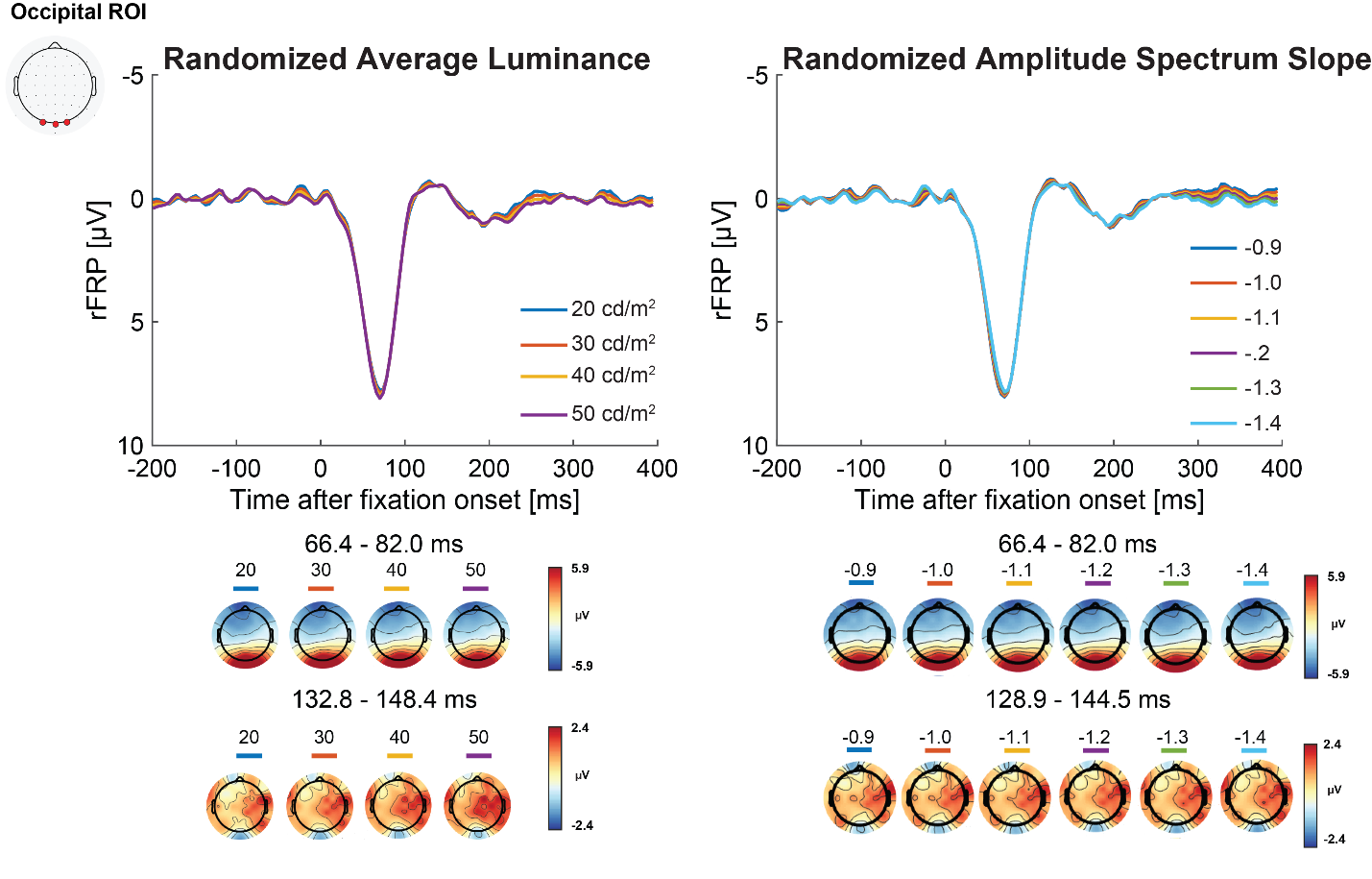


*Note.* The randomized model’s the grand average rFRPs (in microvolts) for each randomized image statistic predictor with positive plotted down at the occipital ROI with the corresponding topographical maps averaged across all electrodes for the peak latency for each randomized predictor.

**Figure A2**

*Model 3 Beta Values from the Occipital ROI*

|  | **Lambda Response** | **FRP N1** |
| --- | --- | --- |
| **Beta Values (in microvolts)** | **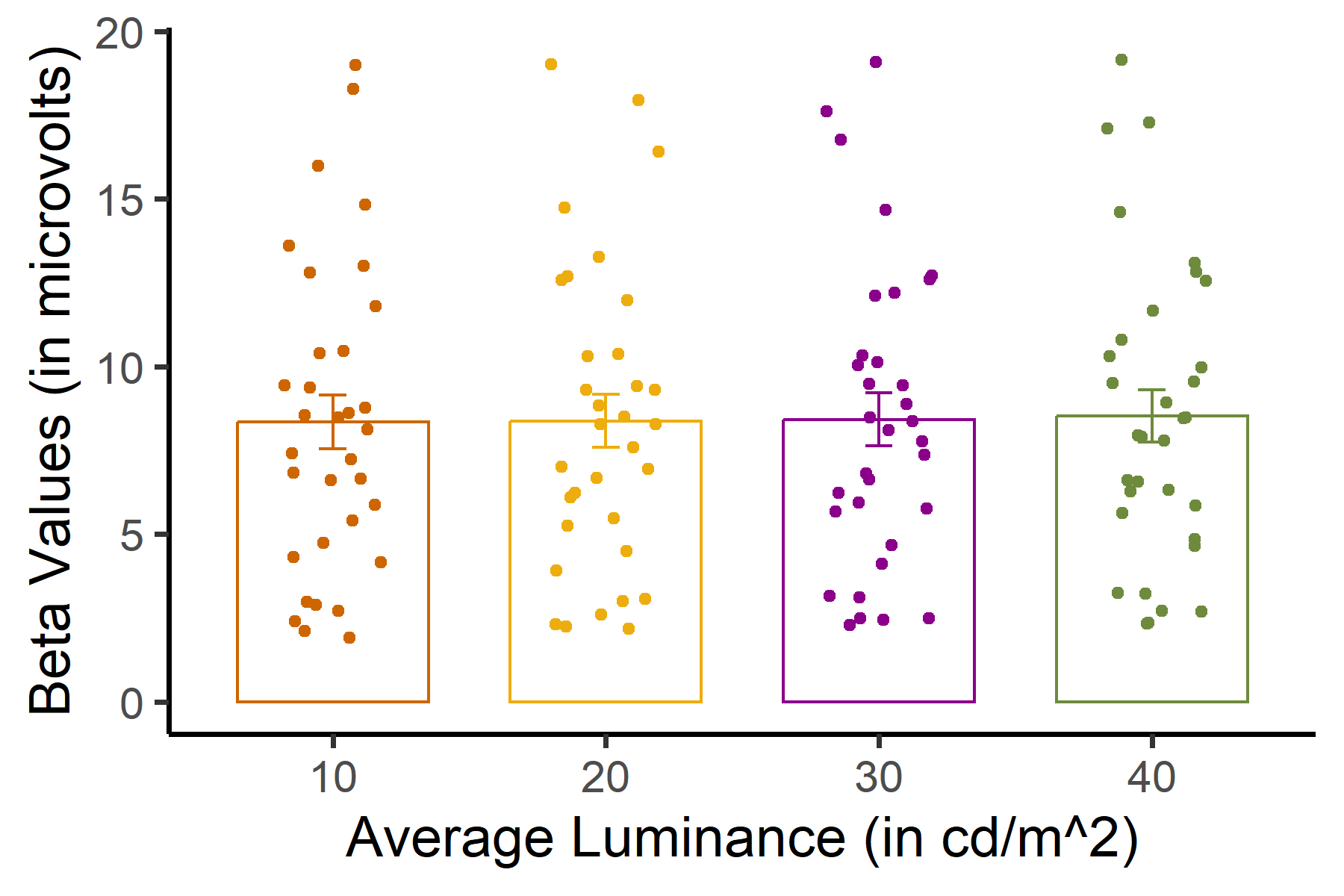** | 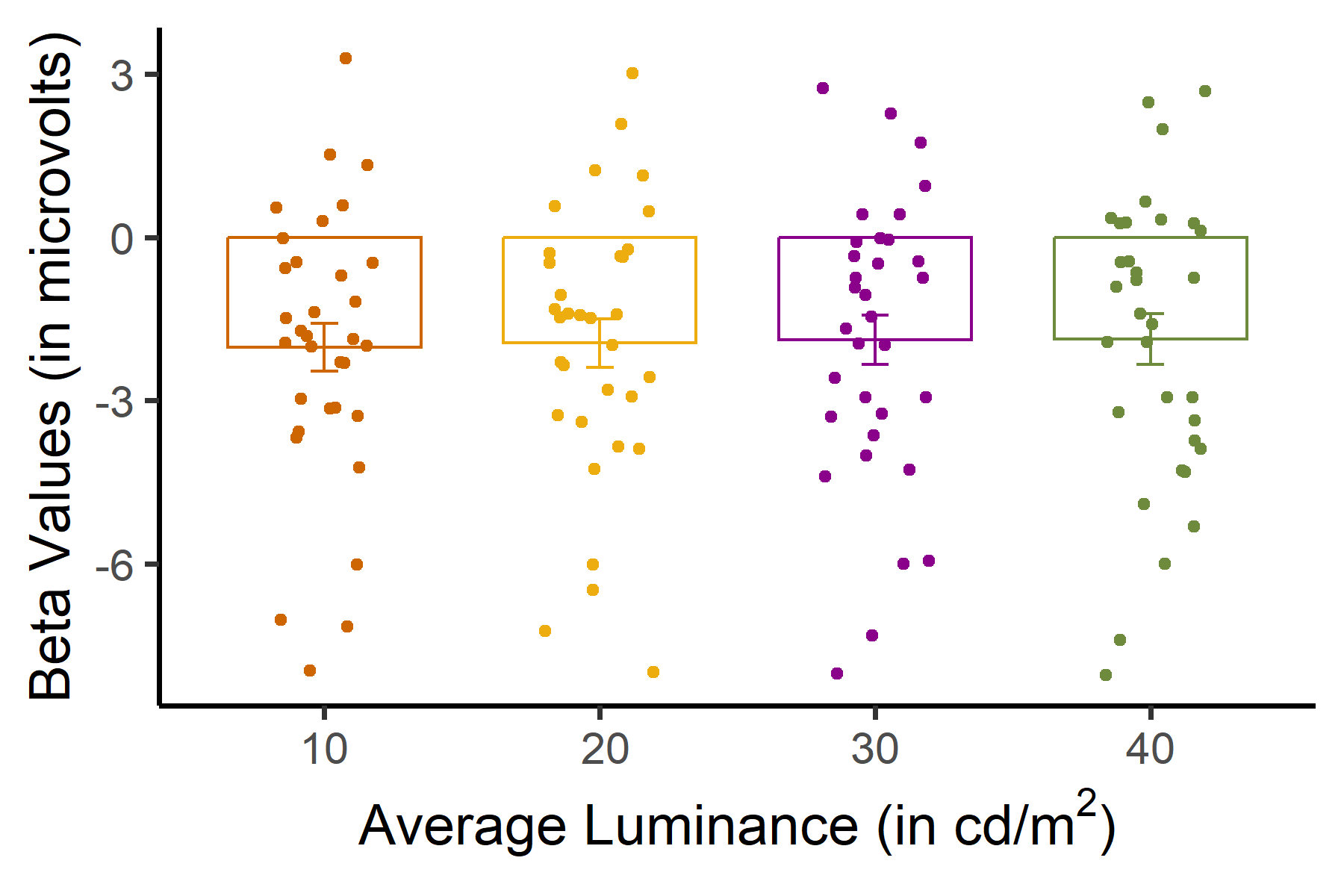 |
| **Randomized Average Luminance (in cd/m^2^)** | | |
| **Beta Values (in microvolts)** | **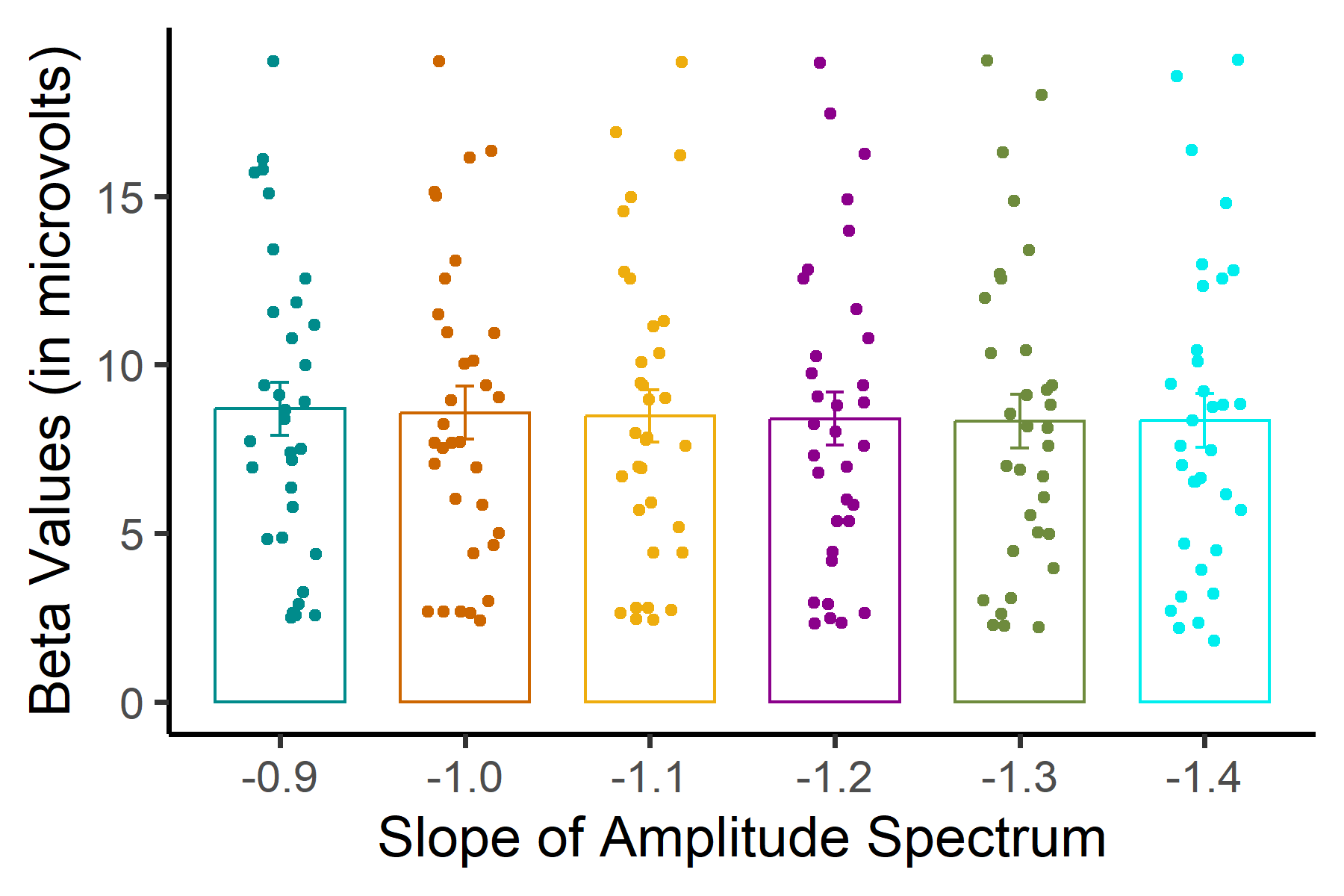** | 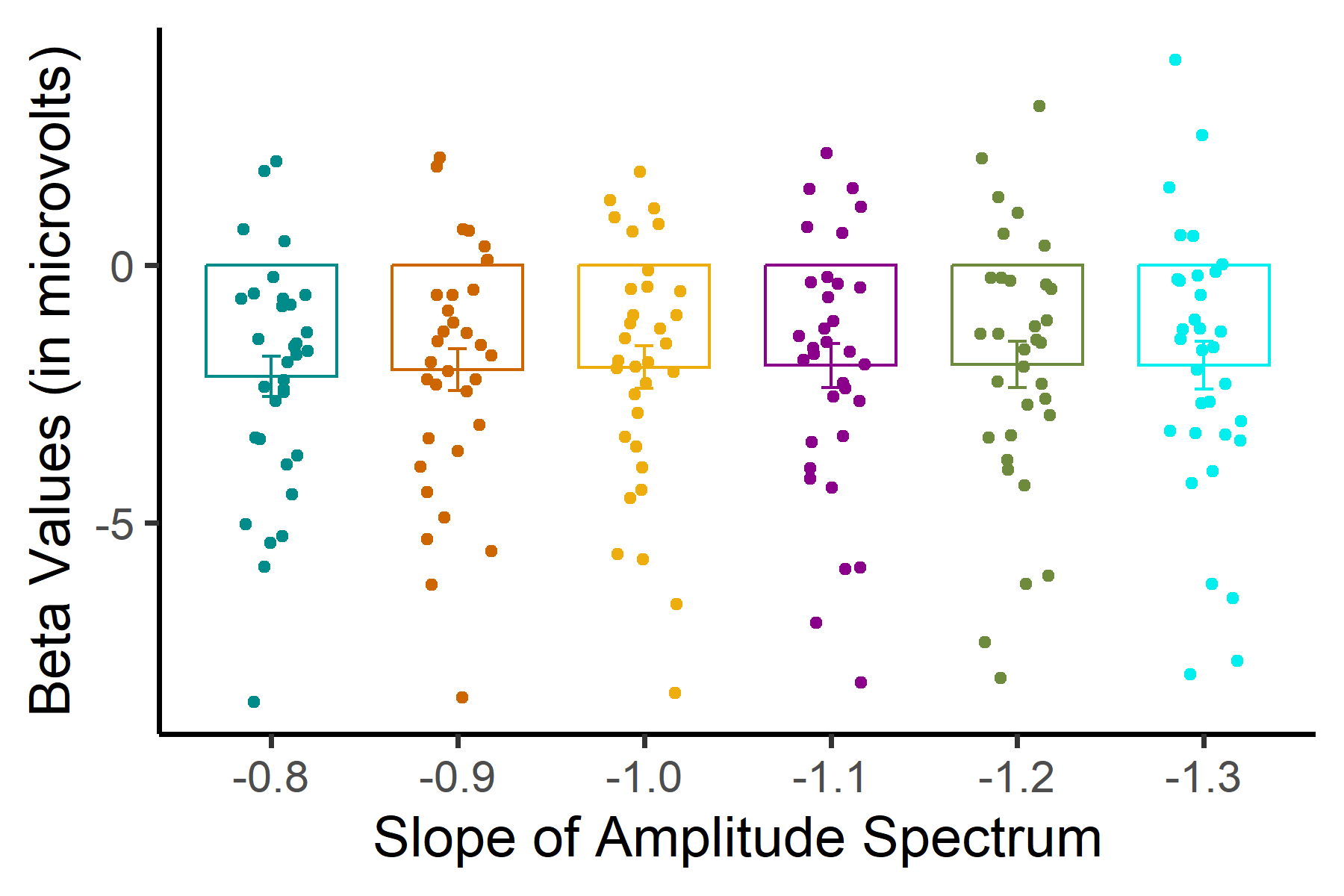 |
| **Randomized Slope of Amplitude Spectrum** | | |

*Note*. This figure shows the betas values for each Model 3­ randomized predictor by FRP component with subject-level variability shown as individual data points and group-level means represented by bar plots with the between-subject standard error.

**Appendix B: Alternative Statistical Approach for Testing Effects of Continuous Predictors**

We ran an additional control analysis to validate the repeated measures approach (presented in the main text) that included using the function: ‘*uf_predictContinuous’* to sample the beta values for the continuous image statistic predictors. This alternative approach to analyzing continuous predictors skips the ‘*uf_predictContinuous’* step in the above-described processing of the data under “Linear Deconvolution Modeling.” This results in different underlying predictor values based on individual distributions instead of having a common predicted value. The resulting beta values for each individual participant were extracted and fit with a linear regression model. We extracted the slope of the linear regression model as an approach to evaluate the impact of the continuous predictors on the rFRP amplitude; a slope of zero would indicate no effect of the predictor. The results using this approach for Models 1 and 2 are consistent overall with the main analysis using a repeated measures approach.

Using the slope testing approach for Model 1, there was a significant effect from a one sample *t* test on the slopes from the linear fit of the beta values associated with the FRP lambda response, t(32) = 2.39, p=0.023, d = 0.42, but not the slopes for the FRP N1 beta values, t(32) = 1.94 , p = 0.062, d = 0.34. For the slope of the amplitude spectrum predictor, there was no effect of the slope of the lambda response beta values, t(32) = 0.55, p =0.59, d = 0.095, but there was a significant effect on the slope of the FRP N1 beta values, t(32) = 3.11, p = 0.0039, d = 0.54.

For Model 2, using one sample *t* test on the slopes from the linear fit of the beta values, there was an effect of luminance on the lambda response beta values, t(32) = 2.20, p = 0.035, d = 0.38, but not on the slope for the FRP N1 beta values, t(32) = 1.57, p = 0.125, d = 0.27. This was again done this for the slope of the amplitude spectrum predictor, where were found an effect on the slope for the lambda response beta values, t(32) = 2,89, p =0.0069, d = 0.50, and the slope for the FRP N1 beta values, t(32) = 2.73, p = 0.010, d = 0.48.
